# Activation of TNF‐α/NF‐κB axis enhances CRL4B^DCAF^ ^11^ E3 ligase activity and regulates cell cycle progression in human osteosarcoma cells

**DOI:** 10.1002/1878-0261.12176

**Published:** 2018-02-20

**Authors:** Caiguo Zhang, Bin Chen, Kaibiao Jiang, Lifeng Lao, Hongxing Shen, Zhi Chen

**Affiliations:** ^1^ Department of Spine Surgery Renji Hospital School of Medicine Shanghai Jiao Tong University China; ^2^ Department of Dermatology University of Colorado Aurora CO USA

**Keywords:** CRL4B E3 ligase, NF‐κB, osteosarcoma, p21^Cip1^, TNF‐α, ubiquitination

## Abstract

Cullin 4B, a member of the Cullins, which serve as scaffolds to facilitate the assembly of E3 ligase complexes, is aberrantly expressed in many cancers, including osteosarcoma. Recently, we observed that CUL4B forms the CRL4B^DCAF^ ^11^ E3 ligase, which specifically ubiquitinates and degrades the cyclin‐dependent kinase (CDK) inhibitor p21^Cip1^ in human osteosarcoma cells. However, the underlying mechanisms regarding the aberrant expression of *CUL4B* and the upstream members of this signaling pathway are mostly unknown. In this study, we demonstrate that nuclear factor kappaB (NF‐κB) is a direct modulator of *CUL4B* expression. The *CUL4B* promoter is responsive to several NF‐κB subunits, including RelA, RelB, and c‐Rel, but not to p50 or p52. Additional studies reveal that the tumor necrosis factor alpha (TNF‐α)/NF‐κB axis pathway is activated in human osteosarcoma cells. This activation causes both CUL4B and NF‐κB subunits to become abundant in the nucleus of human osteosarcoma cells. The down‐regulation of individual genes, including *TNFR1*,* RelA*,* RelB*,* c‐Rel*, and *CUL4B*, or pairs of them, including *TNFR1 *+* RelA*,*TNFR1 *+* RelB*,*TNFR1 *+* c‐Rel*, and *RelA*+*CUL4B*, has similar effects on cell growth inhibition, colony formation, cell invasion, and *in vivo* tumor formation, whereas the overexpression of *CUL4B* in these knockdown cells significantly reverses their phenotypes. The inhibition of the TNF‐α/NF‐κB pathway greatly attenuates CRL4B^DCAF^ ^11^ E3 ligase activity and causes the accumulation of p21^Cip1^, thereby leading to cell cycle arrest at the S phase. Taken together, our results support a model in which the activation of the TNF‐α/NF‐κB axis contributes to an increase in CRL4B^DCAF^ ^11^ activity and a decrease in p21^Cip1^ protein levels, thereby controlling cell cycle progression in human osteosarcoma cells.

**Abbreviations**
BMP: bone morphogenetic protein
CDK: cyclin‐dependent kinase
CRL: Cullin‐RING ubiquitin ligases
DCAF: DDB1‐ and CUL4‐associated factor
DDB1: DNA damage binding protein 1
GSK‐3β: glycogen synthase kinase‐3β
NF‐κB: nuclear factor kappa‐light‐chain‐enhancer of activated B cells
RBX1: RING‐box protein 1
RIP: receptor‐interacting protein
TNF‐α: tumor necrosis factor alpha
TRADD: tumor necrosis factor receptor type 1‐associated DEATH domain protein

## 1. Introduction

All eukaryotes, ranging from bacteria to human cells, encode a family of conserved proteins called Cullins, which commonly function as scaffolds that associate with RING proteins, adaptor proteins, and substrate recognition receptors to form Cullin‐RING ubiquitin ligases (CRLs) (Chen *et al*., 2015; Petroski and Deshaies, 2005; Zhang and Zhang, 2015). Genetic and molecular studies in several model organisms have demonstrated that CRLs recognize a large number of substrates and therefore affect a wide range of biological processes such as cell development, DNA damage and repair, and cell cycle progression (Sang *et al*., 2015; Zhao and Sun, 2013). The human genome contains six classic Cullin members, CUL1, CUL2, CUL3, CUL4A, CUL4B, and CUL5, which are characterized by a Cullin homology (CH) domain in the carboxyl (C)‐terminal, and two atypical Cullins, CUL7 and CUL9, with additional homology domains such as DOC1 (destruction of cyclin B) (Chen *et al*., 2015; Sarikas *et al*., 2011). Interestingly, it appears that the dysregulation of Cullins, especially the six classic Cullins, contributes to tumorigenesis through multiple mechanisms, including cell cycle progression arrest, disrupted DNA damage repair, the attenuated ubiquitination of oncoproteins, and the increased ubiquitination of tumor suppressors (Chen *et al*., 2015; Sarikas *et al*., 2011). Of the eight Cullins, the underlying mechanisms of CUL4A in the pathogenesis of cancer are most characterized. CUL4A is broadly expressed in many cancer types, including breast cancer and hepatocellular carcinoma (Chen *et al*., 1998; Yasui *et al*., 2002). Generally, CUL4A interacts with DNA damage binding protein 1 (DDB1) at the N‐terminal and with RING‐box protein 1 (RBX1) at the C‐terminal; DDB1 further binds to DDB1‐ and CUL4‐associated factors (DCAFs) (Chen *et al*., 2015; Zimmerman *et al*., 2010). The CRL4A complexes target a large number of substrates, including CDK inhibitor 1, or p21^Cip1^ (Hall *et al*., 2014); CDK inhibitor 1B, or p27^Kip1^ (Higa *et al*., 2006); DDB2 (Fischer *et al*., 2011); chromatin licensing and DNA replication factor 1 (CDT1) (Higa *et al*., 2003); signal transducer and activation of transcription 1 (STAT1) (Precious *et al*., 2005; Sharma and Nag, 2014); and checkpoint kinase 1 (Chk1) (Huh and Piwnica‐Worms, 2013). Additionally, the complexes play important roles in different biological processes during tumorigenesis. The amino acid sequence of CUL4B is largely homologous to that of CUL4A (82%); however, they do not exhibit functional redundancy, because the loss of function or mutation of either causes serious developmental defects or diseases (Chen *et al*., 2017). In recent years, growing evidence indicates that CUL4B is highly expressed in several cancer types, such as esophageal cancer and osteosarcoma (Chen *et al*., 2014; Hu *et al*., 2012). Our recent study suggests that CUL4B associates with DDB1, RBX1, and DCAF11 to form a unique CRL4B^DCAF11^ E3 complex in human osteosarcoma cells, and this E3 ligase can specifically target p21^Cip1^ for degradation, thereby regulating cell cycle progression (Chen *et al*., 2017).

Nuclear factor kappa‐light‐chain‐enhancer of activated B cells (NF‐κB), a transcription factor complex containing RelA, RelB, c‐Rel, p50, and p52 subunits, is constitutively active and translocated from the cytoplasm to the nucleus in many cancerous cells such as breast, colon, and prostate cancer cells (Gupta *et al*., 2010; Hoesel and Schmid, 2013). Current views recognize that a number of stimuli, including reactive oxygen species (ROS), tumor necrosis factor alpha (TNF‐α), interleukin 1‐beta (IL‐1β) and bacterial lipopolysaccharides (LPS), are able to activate NF‐κB and its downstream events (Gupta *et al*., 2010; Hoesel and Schmid, 2013). In several cancers, the conserved TNF‐α/NF‐κB axis signaling pathway has been well characterized. The activation of TNF‐α signaling facilitates the TNF receptor (TNFR) to interact with tumor necrosis factor receptor type 1‐associated DEATH domain protein (TRADD), which recruits TNF receptor‐associated factor 2 (TRAF2) and receptor‐interacting protein (RIP) (Wang *et al*., 2000). TRAF2 further recruits IκB kinase (IKK), enabling its activation by RIP. The activated IKK phosphorylates IκBα, an inhibitory protein that binds to NF‐κB and inhibits its translocation, and subsequently degrades it, releasing NF‐κB, which further translocates to the nucleus and mediates the transcription of a large number of genes involved in tumorigenesis (Wang *et al*., 2000). Interestingly, the NF‐κB pathway is activated and is widely involved in osteosarcoma cell proliferation and differentiation by affecting bone morphogenetic protein (BMP) signaling or the glycogen synthase kinase‐3β (GSK‐3β) pathway (Tang *et al*., 2012).

Although we have identified a downstream target of the CRL4B E3 ligase, two critical issues remain obscure: (1) the underlying mechanisms of *CUL4B* overexpression and how they differ from those of other Cullins and (2) the upstream signaling of CUL4B. To address the first issue, we analyzed the promoter sequences of the *Cullin* genes, and we found that the *CUL4B* promoter has an NF‐κB transcription factor‐binding site, GGGGTTTCCC, which was not found in the other *Cullin* genes. Then, we determined that three NF‐κB subunits, RelA, RelB, and c‐Rel, were able to bind to the promoter region of *CUL4B*, but p50 and p52 did not bind. Importantly, TNF‐α/NF‐κB signaling is activated in osteosarcoma cells, increasing the abundance of NF‐κB subunits in the nucleus, thereby activating the expression of *CUL4B* and regulating the ubiquitination of p21^Cip1^. Thus, we answered the two key questions, and our results reveal the important role of the TNF‐α/NF‐κB axis in the regulation of *CUL4B* expression and cell cycle progression in human osteosarcoma cells.

## 2. Materials and methods

### 2.1. Cell lines, culture conditions, and transfection

The human osteoblast cell line hFOB1.1.9 and osteosarcoma cell lines including U2OS, MG63, Saos‐2, and HOS were obtained from the American Type Culture Collection (ATCC, Manassas, VA, USA). The human osteoblast cell lines HOB and Ho‐f were purchased from Sigma (St. Louis, MO, USA) and ScienCell (Carlsbad, CA, USA), respectively. The other cell lines including the pancreatic adenocarcinoma cell line CFPAC‐1, the lung cancer cell line H1299, the breast cancer cell line MCF‐7, the carcinoma cell line Fadu, and the melanoma cell line A375 were purchased from ATCC. All cells were grown in DMEM supplemented with 10% fetal bovine serum (FBS) and 0.1% penicillin/streptomycin and incubated at 37 °C with 5% CO_2_.

The specific knockdown of genes with siRNA or shRNA was performed as previously described (Chen *et al*., 2017). For siRNA or plasmid transfection, cells at approximately 80% confluence were treated with 0.25% Trypsin‐EDTA (Thermo Fisher Scientific, Waltham, MA, USA) and then transfected using Lipofectamine 2000 (Invitrogen, Carlsbad, CA, USA) in suspension with 200 ng of plasmid or siRNA. The transfected cells were immediately plated into 12‐well plates supplemented with 1.0 mL of DMEM and incubated at 37 °C for 48 h. For shRNA‐related transfection, lentiviruses containing either control shRNA or specific shRNA against RelA, RelB, c‐Rel, CUL4B, or TNFR1 were transfected into U2OS cells according to the standard procedure (Chen *et al*., 2017; Zhang *et al*., 2017). The virus‐infected cells were incubated at 37 °C for 24 h and then supplemented with puromycin (1 μg·mL^−1^) for selection. After 48 h, the puromycin‐resistant cells were used for experiments. The siRNA and shRNA information is listed in Table S1.

### 2.2. Human clinical sample collection

Human clinical tissue samples were collected from 54 osteosarcoma patients who were diagnosed with osteosarcoma of different MSTS stages (I‐IV) based on histopathological features at the Department of Spine Surgery, Renji Hospital, School of Medicine, Shanghai Jiao Tong University, Shanghai, China. The basic information for these patients is summarized in Table S2. After surgery, all samples were immediately frozen in liquid nitrogen and transferred to a −80 °C freezer for storage until the time of experiments. All samples were obtained with the agreement of participants following protocols approved by the ethical board of Shanghai Jiao Tong University. All experimental procedures related to human clinical samples were strictly conducted according to the guidelines of the Ethics Committee of Shanghai Jiao Tong University.

### 2.3. Quantitative RT‐PCR analysis

Total RNA was extracted from cells or human clinical samples with TRIzol reagent (Invitrogen) according to the manufacturer's protocol. A dynabeads mRNA purification kit (ThermoFisher Scientific) was used to isolate mRNA from total RNA. Then, 500 ng mRNA was used as the template to synthesize first‐strand cDNA with oligo(dT) primers and M‐MLV reverse transcriptase (Promega, Madison, WI, USA). The resulting cDNA species were subjected to quantitative RT‐PCR (qRT‐PCR) analysis using the Bio‐Rad SYBR Green Master Mix kit (Bio‐Rad, Hercules, CA, USA). All experiments were repeated three times, and gene expression was measured using the 2^−ΔΔCt^ method by normalizing to β*‐Actin*, as previously described (Zhang *et al*., 2014). Primers were described previously (Chen *et al*., 2017).

### 2.4. Western blot analysis

Western blot (WB) analysis was performed as previously described (Chen *et al*., 2017). In brief, cells were lysed and sonicated in 1 × RIPA buffer (Thermo Scientific) supplemented with 50 mm NaF, 1 mm Na_3_VO_4_, and 1 ×  complete protease inhibitor cocktail (Roche, Indianapolis, IN, USA). The protein concentration of whole cell lysates was determined using the Coomassie Plus Protein Assay Kit (Thermo Fisher Scientific). Equal amounts of proteins were denatured at 95 °C for 5 min and then resolved on an SDS/PAGE gel. The GAPDH and Cullin antibodies were described previously (Chen *et al*., 2017; Zhang *et al*., 2017); antibodies against NF‐kB subunits, including anti‐RelA (Cat#: ab16502), anti‐RelB (Cat#: ab180127), anti‐c‐Rel (Cat#: ab133251), anti‐p50 (Cat#: ab32360), and anti‐p52 (Cat#: ab125611), were obtained from Abcam (rabbit, Cambridge, MA, USA). The other primary antibodies used in this study were anti‐TNFR1 (Cat#: ab19139, rabbit, Abcam), anti‐TRADD (Cat#: T9204, rabbit, Sigma), anti‐RIP (Cat#: SAB1400734, rabbit, Sigma), anti‐TRAF2 (Cat#: ab126758, rabbit, Abcam), anti‐IKK (Cat#: ab32041, rabbit, Abcam), anti‐IκBα (Cat#: SAB1305978, rabbit, Sigma), and anti‐pIκBα (Cat#: SAB4300125, rabbit, Sigma). The western blot signals were detected using enhanced chemiluminescence reagents (GE Healthcare, Chicago, IL, USA) with a ChemiDoc MP instrument (Bio‐Rad). The images represent three independent replicates.

### 2.5. Chromatin immunoprecipitation assay

The lentiviruses containing shRNA species, including negative control shRNA or *shRelA* (TRCN0000353629), *shRelB* (TRCN0000014717), *shc‐Rel* (TRCN0000014717), *shp50* (TRCN0000006521), or *shp52* (TRCN0000356047), were transfected into U2OS cells using standard procedures. After transfection for 24 h, the virus‐infected cells were washed with 1 x PBS at room temperature and then crosslinked with 1% formaldehyde for 15 min. The crosslinking reaction was stopped by the addition of glycine to a final concentration of 0.125 m. Cells were then washed twice with 1 × PBS and lysed in hypotonic buffer containing 1% NP‐40, 50 mm NaCl, 10 mm Tris (pH 8.0), 1 mm DTT, 2 mm EDTA, and 1 x proteinase inhibitor, sonicated for 2 min, and centrifuged (13 000 ***g*** for 10 min at 4 °C). A total of 50 μL supernatant was removed as INPUT, and the remnant was incubated with Protein A–Sepharose beads (Sigma) and specific antibodies overnight at 4 °C. Beads were washed five times with buffer containing 0.1% SDS, 0.5% Triton X‐100, 20 mm Tris, 150 mm NaCl, 1 mm DTT, 2 mm EDTA, and 1 x proteinase inhibitor and then with TE buffer. After an extensive wash step, the complexes were eluted with buffer containing 1 mm sodium bicarbonate and 1% SDS. DNA was purified using the QIAquick PCR purification kit (Qiagen, Germantown, MD, USA). PCR was performed with primers described previously (Chen *et al*., 2017).

### 2.6. Luciferase assay

Cells expressing NF‐kB subunits, including *pCDNA3‐RelA‐Flag*,* pCDNA3‐RelB‐Flag*,* pCDNA3‐c‐Rel‐Flag*,* pCDNA3‐p50‐Flag*, or *pCDNA3‐p52‐Flag*, were co‐transfected with the firefly luciferase reporter vector *pGL4.26‐P*_*CUL4B‐WT*_ *‐Luc* or *pGL4.26‐P*_*CUL4B‐Mutant*_ *‐Luc* and the Renilla luciferase vector pRL‐TK. After incubation at 37 °C for 24 h, cells were subjected to luciferase assay using a Dual Luciferase Reporter Assay System (Promega) according to the manufacturer's protocol. The relative luciferase activity was determined by normalizing firefly luciferase to Renilla luciferase.

### 2.7. Immunofluorescence and immunohistochemistry

For Immunofluorescence (IMF) staining, cells cultured on glass coverslips were fixed with 4% paraformaldehyde (PFA) for 30 min at room temperature and then washed with 1 x PBS three times. Cells were blocked with 1% BSA for 30 min at room temperature and then incubated with primary antibodies including anti‐RelA (Cat#: ab16502), anti‐RelB (Cat#: ab180127), anti‐p52 (Cat#: ab125611), and anti‐CUL4B (Cat#: SAB1406670, mouse, Sigma) for 1 h at room temperature. After an extensive wash step, cells were incubated with the secondary antibodies for 1 h at room temperature. Finally, cells were counterstained with DAPI. Immunohistochemistry (IHC) assays were performed as previously described (Huang *et al*., 2013). Tissue samples were fixed in 10% neutral‐buffered formalin, embedded in paraffin, and sectioned from the midline of samples. Five‐micrometer‐thick sections were deparaffinized, and antigens were unmasked and immunohistochemically stained for CUL4B. Images were captured using a Leica DM 500B fluorescence microscope.

### 2.8. Cell cycle progression by flow cytometry

Cell cycle analysis was performed as previously described (Chen *et al*., 2016). Briefly, trypsin/EDTA‐treated cells were fixed with 70% ethanol at 4 °C for 24 h and then stained with a buffer containing 50 μg·mL^−1^ propidium iodide (PI), 50 μg·mL^−1^ RNase, 0.1% Triton X‐100, 50 μg·mL^−1^ RNase, and 0.1 mm EDTA at 37 °C for 30 min. Cell cycle distribution was analyzed by flow cytometry (BD Biosciences, San Jose, CA, USA). The cells in the G1, S, and G2/M phases were counted. All samples were tested in triplicate.

### 2.9. Cell proliferation, colony formation, and cell invasion assays

For cell proliferation assays, cells were grown in 96‐well plates, and cell viability was determined every 24 h using an MTT kit (Roche) according to the manufacturer's protocol. For colony formation assays, cells were seeded into 24‐well plates at a density of 1000 cells per well and then continuously cultured in serum‐free DMEM medium for 14 days with a medium change every 3 days. Cell colonies were fixed with 4% paraformaldehyde fixative (PFA) for 30 min and then stained for 20 min with a buffer containing 25% methanol and 0.5% crystal violet. Cells were washed with ddH_2_O five times to remove free dye. For invasion assays, cells were suspended in serum‐free DMEM at 6 × 10^5^ cells·μL^−1^. A total of 500 μL of DMEM with 10% FBS was placed in the lower chamber, and 200 μL of the cell suspension was added to the upper chamber coated with a layer of membrane matrix solution. After incubation at 37 °C for 48 h, the noninvaded cells and media were gently removed with cotton wool, and the cells on the lower surface were fixed in methanol, stained with 0.1% crystal violet, and imaged. All experiments were performed in triplicate.

### 2.10. *In vivo* tumor formation assay

Six‐week‐old female athymic *nu*/*nu* mice (Shanghai SLAC Laboratory Animal Co. Ltd., Shanghai, China) were maintained in accordance with the guidelines of the Institutional Animal Care and Use Committee (IACUC) of Shanghai Jiao Tong University. A total of 1 × 10^6^ U2OS cells with knocked down RelA, RelB, c‐Rel, TNFR1, CUL4B, TNFR1 + RelA, TNFR1 + RelB, TNFR1 + c‐Rel, or CUL4B+RelA and U2OS cells overexpressing CUL4B in a TNFR1‐KD, TNFR1‐KD + RelA‐KD, TNFR1‐KD + RelB‐KD, or TNFR1‐KD + c‐Rel‐KD background were suspended in 100 μL of Matrigel Matrix (BD Biosciences) and diluted 1:1 with PBS. The cell suspension was injected intradermally into the flank of mice (two tumors per mouse, five mice per group). Tumor volume was measured with fine calipers at 5‐day intervals and calculated with the following formula: Volume* = *(Length* × *Width^2^)/2.

### 2.11. *In vivo* ubiquitination analysis

The *in vivo* ubiquitination analysis was performed as previously described (Chen *et al*., 2017). Briefly, cells co‐expressing HA‐ubiquitin and Flag‐tagged CUL4B were incubated at 37 °C for 48 h and then lysed with a buffer containing 10 mm Tris/HCl (pH 8.0), 150 mm NaCl, and 2% SDS. After centrifugation at 13 000 ***g*** for 15 min, the supernatant was incubated with anti‐Flag‐Agarose beads at 4 °C for 3 h, and the beads were then washed with lysis buffer five times. The Flag‐associated protein complex was denatured at 95 °C for 5 min and then resolved on an SDS/PAGE gel. The ubiquitination of p21 was determined by immunoblotting with an anti‐HA antibody.

### 2.12. Statistical analysis

All experiments in this study were independently replicated at least three times. Statistical analyses were performed using spss version 22 software (North Castle, NY, USA) and two‐sided Student's *t*‐tests. Significance was set at *P *<* *0.05.

## 3. Results

### 3.1. CUL4B is overexpressed in human osteosarcoma cells and tissues

We have previously shown that CUL4B, but not the other Cullins, is up‐regulated in human osteosarcoma cells (Chen *et al*., 2017). In this study, we further confirmed this observation by examining the expression of *Cullins* in the four osteosarcoma cell lines U2OS, MG63, Saos‐2, and HOS and in the three osteoblast cell lines hFOB1.19, HOB, and Ho‐f. As shown in Fig. S1A, *CUL4B* mRNA was significantly up‐regulated (~fourfold induction) in all four osteosarcoma cell lines compared to osteoblast cells. Additionally, the expression of *CUL4A*, a paralogue of *CUL4B*, was slightly increased (~1.5‐fold induction) in osteosarcoma cells compared to osteoblast cells. However, no obvious changes were observed for the other *Cullins*. Based on the similar levels of *CUL4B* in these three osteoblast cell lines, we only used hFOB1.19 cells as control in subsequent experiments. To assess whether *CUL4B* overexpression is associated with osteosarcoma tumorigenesis, we collected 54 paired cancerous tissues and adjacent nontumor tissues. The detailed information for these patients is summarized in Table S2. Compared to levels in healthy tissues, we found 12 cancerous tissues that showed decreased *CUL4B* levels and 42 cancerous tissues that had increased *CUL4B* levels (Fig. S1B). Moreover, the increased expression of *CUL4B* in osteosarcoma patients was associated with larger tumor size (≥ 12 cm, *P* = 0.003) (Fig. S1C) and higher MSTS stage (*P *= 0.0001) (Fig. S1D). However, the expression of *CUL4B* had no significant correlation with other parameters including age and gender (Table S2). Thus, the significant overexpression of *CUL4B* was frequently observed in the majority of the osteosarcoma tumors.

To further determine whether only *CUL4B* was overexpressed in osteosarcoma tumors, we randomly selected four paired tissues from patients who were diagnosed with osteosarcoma at different MSTS stages (I‐IV, one paired tissue per stage) based on the histopathological features and measured the expression of *Cullins* in these samples. As shown in Fig. S2A, *CUL4B* mRNA levels were positively correlated with the malignancy levels of the osteosarcoma patients, that is, the lower the degree of malignancy, the lower the expression of *CUL4B*; correspondingly, the more severe the degree of malignancy, the higher the expression of *CUL4B*. The expression levels of the other *Cullins* were similar to those in osteosarcoma cells; that is, *CUL4A* had a slight induction in samples from MSTS stage II‐IV (~1.5‐ to twofold), whereas *CUL1*,* CUL2*,* CUL3*,* CUL5*, and *CUL7* showed no obvious changes in cancerous tissues from patients who were diagnosed with MSTS stage IV (Fig. S2A). In addition, we examined Cullin protein levels in these four paired tissue samples. Consistent with mRNA levels, we observed a significant increase of CUL4B protein levels and a slight induction of CUL4A in cancerous samples from patients in MSTS stages III and IV but no induction of the other Cullin members (Fig. S2B). Next, we analyzed the histological changes of CUL4B using IHC staining in these four paired tissue samples. As expected, CUL4B staining was more obvious in cancerous tissues than in nontumor tissues (Fig. S2C). Given that the mRNA and protein levels of CUL4B were up‐regulated in osteosarcoma cells and cancerous samples, we speculated that the increase of CUL4B protein level resulted from its mRNA induction. These results suggested that a transcriptional mechanism might exist to regulate *CUL4B* overexpression in osteosarcoma cells.

### 3.2. Transcription factor NF‐κB specifically binds to the CUL4B promoter

**Figure 1:**
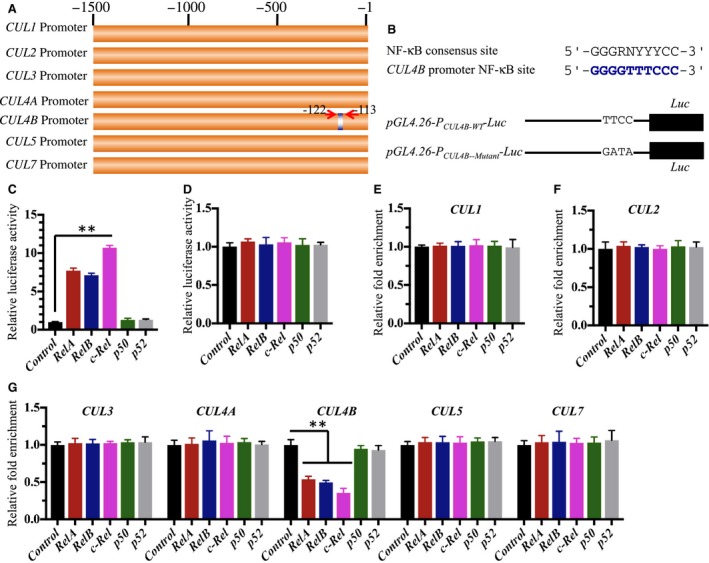
NF‐κB regulates CUL4B expression in human osteosarcoma cells. (A) Schematic diagrams of the Cullin gene promoters. The promoter regions (−1 to −1500) of *CUL1*,*CUL2*,*CUL3*,*CUL4A*,*CUL4B*,*CUL5*, and *CUL7* were indicated, and the NF‐κB binding site in the *CUL4B* promoter was indicated by blue color. (B) Consensus NF‐κB binding site sequence in the *CUL4B* promoter. The consensus sequence of NF‐κB in the *CUL4B* promoter was indicated with blue color, and the *pGL4.26‐* *P*_*CUL*__*4B*_ *‐Luciferase* and its mutant version were indicated. (C‐D) Luciferase assay. U2OS cells expressing *pCDNA3‐RelA‐Flag*,*pCDNA3‐RelB‐Flag*,*pCDNA3‐c‐Rel‐Flag*,*pCDNA3‐p50‐Flag*, and *pCDNA3‐p52‐Flag* were co‐transfected with the firefly luciferase reporter vector *pGL4.26‐* *P*_*CUL*__*4B‐*_ _*WT*_ *‐Luc* or *pGL4.26‐* *P*_*CUL*__*4B‐Mutant*_ *‐Luc* and the Renilla luciferase vector pRL‐TK. After incubation at 37 °C for 24 h, the cells were subjected to luciferase assays using a Dual Luciferase Reporter Assay System. (E‐G) ChIP assay. U2OS cells with down‐regulated *RelA*,* RelB*,* c‐Rel*,* p50*, or *p52* were subjected to the ChIP assay using specific antibodies, and the binding to Cullin genes was measured by qRT‐PCR. The enrichment of each *Cullin* gene was normalized against the control vector. **P <* *0.001.

To illuminate the underlying mechanisms of *CUL4B* overexpression in human osteosarcoma cells, we analyzed the promoter regions of the *Cullin* genes. Accordingly, we selected a 1500‐bp promoter region of each *Cullin* gene and searched the transcription factor‐binding sites of these sequences in the ALGGEN‐PROMO database (http://alggen.lsi.upc.es/cgi-bin/promo_v3). By comparing the potential transcription factor‐binding sites, we found that the *CUL4B* promoter region contained an NF‐κB consensus site (GGGGTTTCCC) located at −113 to −122 bp from the initiation site (Fig. 1A), whereas the promoter regions of other *Cullins* did not contain the NF‐κB consensus site (Fig. 1A). Based on previous experimental results and our findings that *CUL4B* was overexpressed in osteosarcoma cells and cancerous tissues, we speculate that NF‐κB can specifically bind to the *CUL4B* promoter region and regulate its expression. To evaluate this hypothesis, we generated the individual NF‐κB subunit overexpression constructs and *CUL4B* promoter luciferase reporter constructs containing either wild‐type (WT) or mutated NF‐κB binding sites (Fig. 1B). After co‐transfecting the individual NF‐κB subunit overexpression vectors with *pGL4.26‐P*_*CUL4B‐WT*_ *‐Luc* or *pGL4.26‐P*_*CUL4B‐Mutant*_ *‐Luc* and the Renilla luciferase vector pRL‐TK, we investigated the contributions of the individual NF‐κB subunits in the activation of *CUL4B*. As shown in Fig. 1C, among the five NF‐κB subunits, only *RelA*,* RelB*, and *c‐Rel* activated the *CUL4B* promoter, and the strongest effect was observed with *c‐Rel* (~11‐fold). Similar effects were observed with *RelA* and *RelB* (~sevenfold to eightfold). In contrast, none of the NF‐κB subunits activated the mutated *CUL4B* promoter construct (Fig. 1D). These results suggested that *RelA*,* RelB*, and *c‐Rel*, but not *p50* and *p52*, were able to modulate the expression of *CUL4B*.

To understand and verify the transcriptional regulation of *CUL4B* by NF‐κB subunits, we knocked down the individual NF‐κB subunits using shRNA species against the subunits and assessed *CUL4B* promoter binding using the Chromatin immunoprecipitation (ChIP) assay. As shown in Fig. S3A, the shRNA species against these NF‐κB subunits significantly down‐regulated their corresponding genes. Compared to the nontargeted control shRNA, we found that the down‐regulation of NF‐κB subunits did not affect binding to *CUL1*,* CUL2*,* CUL3*,* CUL4A*,* CUL5*, or *CUL7* promoters (Fig. 1E–G). However, we observed a significant decrease in *CUL4B* promoter binding after the knockdown of *RelA*,* RelB*, or *c‐Rel*, but not *p50* or *p52* (Fig. 1E–G). In addition, we examined the mRNA and protein levels of Cullin members after the down‐regulation of *RelA*,* RelB*, or *c‐Rel* in both hFOB1.19 and U2OS cells. Interestingly, we observed a significant decrease in CUL4B mRNA and protein levels after the down‐regulation of *RelA*,* RelB*, and *c‐Rel*, but not the other Cullins (Figs S3B–D and S4A–C). These results further indicated the specificity of the effects of *RelA*,* RelB*, and *c‐Rel* in the regulation of *CUL4B* expression in osteosarcoma cells. Based on the similar down‐regulation effects of the two shRNA species, we only selected one shRNA for each gene to knock down its expression in the following experiments.

### 3.3. NF‐κB subunits are activated and abundant in the nucleus in osteosarcoma cells

**Figure 2:**
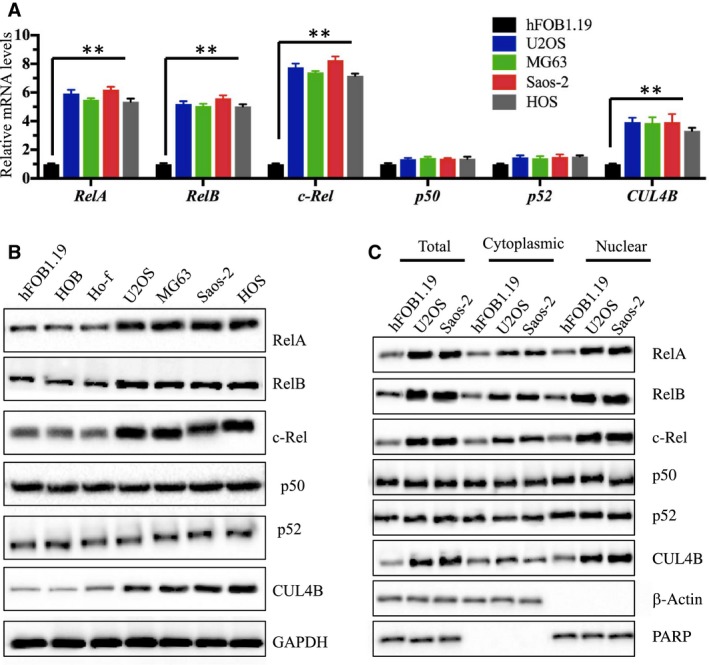
*RelA*,* RelB*, and *c‐Rel* are up‐regulated in human osteosarcoma cells. (A) Expression of NF‐κB subunits in osteosarcoma cells. The mRNA levels of *RelA*,* RelB*,* c‐Rel*,* p50*,* p52*, and *CUL4B* were examined in U2OS, MG63, Saos‐2, and HOS cells by normalizing them against the expression levels in hFOB1.19 cells. **P <* *0.001. (B) The protein levels of NF‐κB subunits in osteosarcoma cells. The protein levels of RelA, RelB, c‐Rel, p50, p52, and CUL4B were examined in hFOB1.19, HOB, Ho‐f, U2OS, MG63, Saos‐2, and HOS cells. GAPDH was used as the loading control. (C) Protein levels of NF‐κB subunits in the cytoplasm and nucleus. The cell lysate (total), cytoplasmic, and nuclear portions of the cells were prepared, and immunoblots were performed to examine RelA, RelB, c‐Rel, p50, p52, and CUL4B levels in these three portions. β‐Actin and PARP were used as controls to determine the levels of cytoplasmic and nuclear proteins, respectively.

Given that *RelA*,* RelB*, and *c‐Rel* bind to the promoter region of *CUL4B* and regulate its expression, thereby causing *CUL4B* overexpression in osteosarcoma cells, we sought to determine whether the NF‐κB subunits were activated. To this end, we primarily examined the expression of NF‐κB subunits in U2OS, MG63, Saos‐2, and HOS cells using hFOB1.19 cells as a control. As shown in Fig. 2A, *RelA*,* RelB*, and *c‐Rel* mRNA species were significantly up‐regulated in all four osteosarcoma cell lines compared to hFOB1.19 cells, with the highest expression being observed in *c‐Rel* (~eightfold), and similar effects were observed in *RelA* and *RelB* (~sixfold). However, no obvious changes were observed in the expression levels of *p50* and *p52* under similar conditions. We then examined the protein levels of these NF‐κB subunits and found the significant inductions of RelA, RelB*,* and c‐Rel proteins but not those of p50 and p52 (Fig. 2B).

NF‐κB subunits predominantly exist in the cytoplasm in the inactive state, and they translocate into the nucleus and bind to target DNA sequences after stimulation by TNF‐α in cancer cells (Karin, 1999). To determine whether NF‐κB subunits translocate from the cytoplasm to the nucleus or are abundant in the nucleus, we isolated the cytoplasmic and the nuclear contents from hFOB1.19, U2OS, and Saos‐2 cells, and examined the NF‐κB subunit protein levels. As shown in Fig. 2C, we observed osteosarcoma cells had increased RelA, RelB, c‐Rel, and CUL4B levels in the cytoplasm and nucleus compared to hFOB1.19 cells, but not p50 and p52. More importantly, these proteins were much more abundant in the nucleus than the cytoplasm in osteosarcoma cells (Fig. 2C). To further verify this observation, we performed IMF staining to evaluate the native protein levels of RelA, RelB, p52, and CUL4B in hFOB1.19 and U2OS cells. Consistent with the western blot results, the IMF results also indicated that RelA, RelB, and CUL4B are much more abundant in the nucleus than the cytoplasm in U2OS cells compared to hFOB1.19 cells, but p52 levels and distribution were similar between the cell lines (Fig. S5). In addition, we also determined the localization patterns of RelA, RelB, p52, and CUL4B in clinical samples from patient 4, who was diagnosed with osteosarcoma at MSTS stage IV based on the histopathological features (as shown in Fig. S2C). The IMF results significantly indicated that RelA, RelB, and CUL4B were dominant in the nucleus in cancerous tissue compared to the noncancerous tissue, but p52 did not exhibit this pattern (Fig. S6). These results suggested that the increased RelA, RelB, and c‐Rel protein levels in the nucleus might bind to the promoter region of *CUL4B* and activate *CUL4B* expression.

### 3.4. Down‐regulation of NF‐κB subunits inhibits the cell proliferation, invasion, colony forming ability, and tumorigenesis of osteosarcoma cells

**Figure 3:**
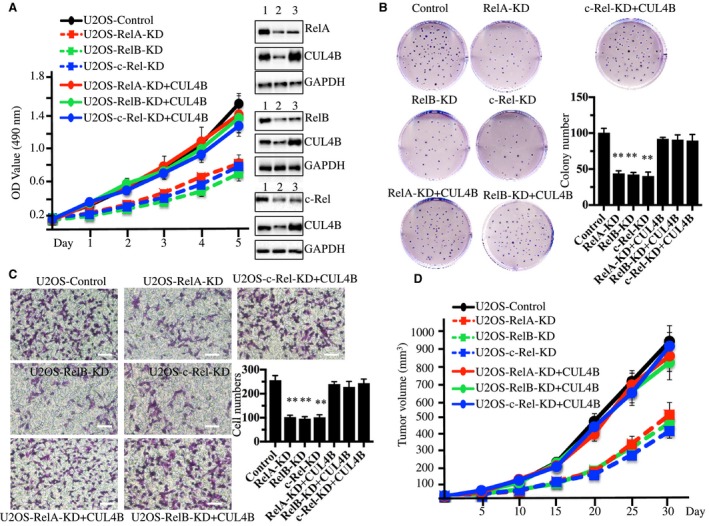
Knockdown of RelA, RelB, or c‐Rel inhibits osteosarcoma cell growth. (A) Knocking down *RelA*,* RelB*, or *c‐Rel* inhibited osteosarcoma cell proliferation. U2OS cells were transfected with Con‐shRNA (U2OS‐Control), RelA‐shRNA (U2OS‐RelA‐KD), RelB‐shRNA (U2OS‐RelB‐KD), c‐Rel‐shRNA (U2OS‐c‐Rel‐KD), RelA‐shRNA + pCDNA3‐CUL4B (U2OS‐RelA‐KD + CUL4B), RelB‐shRNA + pCDNA3‐CUL4B (U2OS‐RelB‐KD + CUL4B), or c‐Rel‐shRNA + pCDNA3‐CUL4B (U2OS‐c‐Rel‐KD + CUL4B). After a 48‐h incubation, MTT assays were conducted to evaluate cell proliferation with absorbance measurement at 490 nm. The knockdown efficiency of RelA, RelB, and c‐Rel was measured with western blots (right panel). 1 (U2OS‐Control), 2 (U2OS‐RelA, RelB, or c‐Rel‐KD), 3 (U2OS‐RelA, RelB, or c‐Rel‐KD + CUL4B). (B) Knocking down *RelA*,* RelB*, and *c‐Rel* decreased colony formation rates. Cells used in A were seeded onto 12‐well plates and cultured with 0.1 mL of fresh medium containing 0.5% FBS for two weeks. **P <* *0.001 (C) Knocking down *RelA*,* RelB*, and *c‐Rel* decreased cell invasion. Cells used in A were subjected to Boyden chamber assays, and the invasive cells were counted (right panel). **P <* *0.001. Bars=100 μm. (D) Knocking down *RelA*,* RelB*, and *c‐Rel* decreased the *in vivo* tumor formation ability. Cells used in A were injected intradermally into the flanks of mice. Tumor volumes were measured with fine calipers at 5‐day intervals.

Our previous results showed that the down‐regulation of *CUL4B* resulted in the inhibition of cell proliferation and a decrease in colony formation (Chen *et al*., 2017). We next sought to determine whether knocking down *RelA*,* RelBI,* and *c‐Rel* would have similar effects. As expected, cell proliferation assays indicated that the down‐regulation of *RelA*,* RelB*, and *c‐Rel* significantly inhibited the growth of U2OS cells (Fig. 3A). The colony formation assay indicated that U2OS cells expressing lower levels of *RelA*,* RelB*, and *c‐Rel* had significantly reduced colony forming ability (Fig. 3B). In addition, the decreased expression of *RelA*,* RelB*, and *c‐Rel* dramatically reduced the invasiveness of U2OS cells (Fig. 3C). To evaluate whether knocking down *RelA*,* RelB*, and *c‐Rel* results in similar suppressive effects *in vivo*, we injected nude mice with *RelA*,* RelB, c‐Rel*, or control shRNA‐transduced U2OS cells and monitored the tumorigenesis for 30 days by measuring tumor volumes at 5‐day intervals. Mice injected with U2OS‐*RelA*‐shRNA, U2OS‐*RelB*‐shRNA, and U2OS‐*c*‐*Rel*‐shRNA cells showed significantly slower tumor growth and decreased tumor volumes (51% reduction by day 25) compared with mice injected with U2OS‐control‐shRNA cells (Fig. 3D). To determine whether the decreased cell proliferation, colony formation ability, invasion, and *in vivo* tumor formation of U2OS cells with down‐regulated NF‐κB subunits were mediated directly through CUL4B or other downstream targets of NF‐κB, we constructed the pCDNA3‐CUL4B vector and transfected it into U2OS‐*RelA*‐shRNA, U2OS‐*RelB*‐shRNA, and U2OS‐*c*‐*Rel*‐shRNA cells and observed whether these phenotypes could be rescued by the overexpression of *CUL4B*. Interestingly, the overexpression of *CUL4B* significantly reversed the decreased cell proliferation, colony formation ability, invasiveness, and *in vivo* tumor formation ability (Fig. 3A–D). Thus, these results clearly demonstrated that *CUL4B* is a downstream target of NF‐κB. A great number of NF‐κB target genes, such as cytokines (e.g., interleukins, IL‐1a, IL‐1b, IL‐2, and IL‐6), chemokines (e.g., macrophage chemotactic protein (MCP‐1) and C‐X‐C motif chemokine ligand 5 (CXCL5)), and immunoreceptors (e.g., C‐C chemokine receptors (CCR5 and CCR7)), have been identified (Pahl, 1999). To determine whether *CUL4B* is specifically regulated by NF‐κB in osteosarcoma cells, we selected IL‐6, CXCL5, and CCR5 as representatives to monitor their expression in U2OS, MG63, Saos‐2, and HOS cells. As shown in Fig. S7, our results indicated that the expression of *IL‐6*,* CXCL5*, and *CCR5* in osteosarcoma cells was similar to their levels in hFOB1.19 cells. Next, we overexpressed *IL‐6* in U2OS cells with knocked down RelA, RelB, or c‐Rel to determine whether its overexpression could rescue diminished cell proliferation and colony formation phenotypes. Our results indicated that *IL‐6* overexpression cannot reverse defects caused by the down‐regulation of RelA, RelB, or c‐Rel in U2OS cells, but CUL4B can rescue these defects (Fig. S8).

### 3.5. Activation of the TNF‐α/NF‐κB axis in human osteosarcoma cells

**Figure 4:**
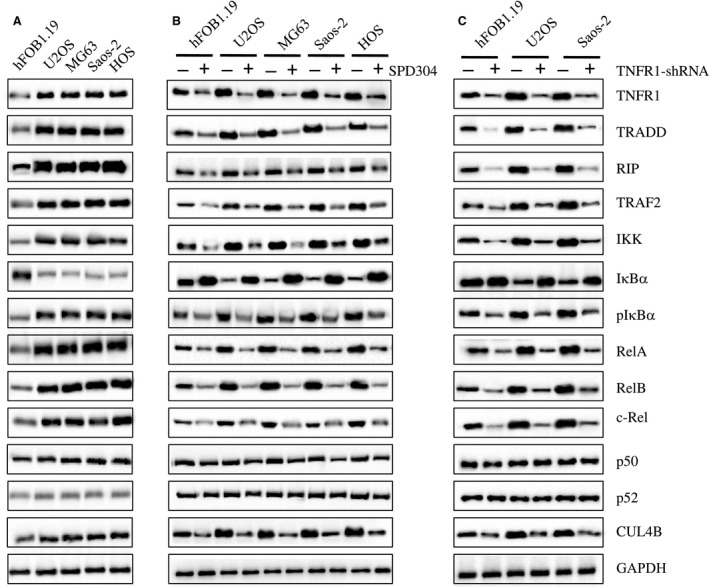
TNF‐α/NF‐κB axis is activated in human osteosarcoma cells. (A) The protein levels of critical members in the TNF‐α/NF‐κB axis. The protein levels of TNFR1, TRADD, RIP, TRAF2, IKK, IκBα, phosphorylated IκBα (pIκBα), RelA, RelB, c‐Rel, p50, p52, and CUL4B were measured in hFOB1.19, U2OS, MG63, Saos‐2, and HOS cells. GAPDH was used as the loading control. (B) Effects of SPD304 on the protein levels of the TNF‐α/NF‐κB axis members. hFOB1.19, U2OS, MG63, Saos‐2, and HOS cells were treated with SPD304 for 24 h, and then, the levels of proteins indicated in (A) were determined by immunoblots. (C) Effects of knocking down KNFR1 on the protein levels of the TNF‐α/NF‐κB axis members. hFOB1.19, U2OS, and Saos‐2 cells were transfected with TNFR1‐shRNA, and the stable cell lines were subjected to immunoblots to determine the TNF‐α/NF‐κB axis member protein levels.

Given that the NF‐κB subunits are often activated by TNF‐α in several cancer types, we sought to examine whether the TNF‐α‐mediated signaling pathway is activated in osteosarcoma cells. To this end, we examined some key proteins, including TNFR1, TRADD, TRAF2, RIP, IKK, and IκBα in the TNF‐α**/**NF‐κB axis. The immunoblot results indicated that TNFR1, TRADD, TRAF2, RIP, and IKK were all activated (Fig. 4A). In contrast, the protein levels of IκBα, an inhibitory protein of NF‐κB, were down‐regulated, whereas the phosphorylation of IκBα was increased (Fig. 4A). These results indicated that the TNF‐α/NF‐κB axis was activated in human osteosarcoma cells. To further verify this conclusion, we treated hFOB1.19, U2OS, MG63, Saos‐2, and HOS cells with SPD304, a cell permeable inhibitor of TNF‐α, to disrupt the TNF‐α−mediated signaling and then determined the levels of NF‐κB subunits and the CUL4B protein. As shown in Fig. 4B, inhibiting TNF‐α signaling not only reduced TNFR1, TRADD, TRAF2, RIP, and IKK protein levels but also decreased RelA, RelB, c‐Rel, and CUL4B protein levels. However, we did not observe any obvious changes in the protein levels of p50 or p52 (Fig. 4B). In addition, we knocked down *TNFR1* using shRNA in hFOB1.19, U2OS, and Saos‐2 cells and determined the effects on its downstream signaling. Similar to the SPD304 treatment, knocking down *TNFR1* decreased TNFR1, TRADD, TRAF2, RIP, IKK, RelA, RelB, c‐Rel, and CUL4B protein levels (Fig. 4C). These results clearly indicated that the TNF‐α/NF‐κB axis was activated in human osteosarcoma cells.

### 3.6. Inhibition of the TNF‐α/NF‐κB axis attenuates CRL4B E3 ligase activity

**Figure 5:**
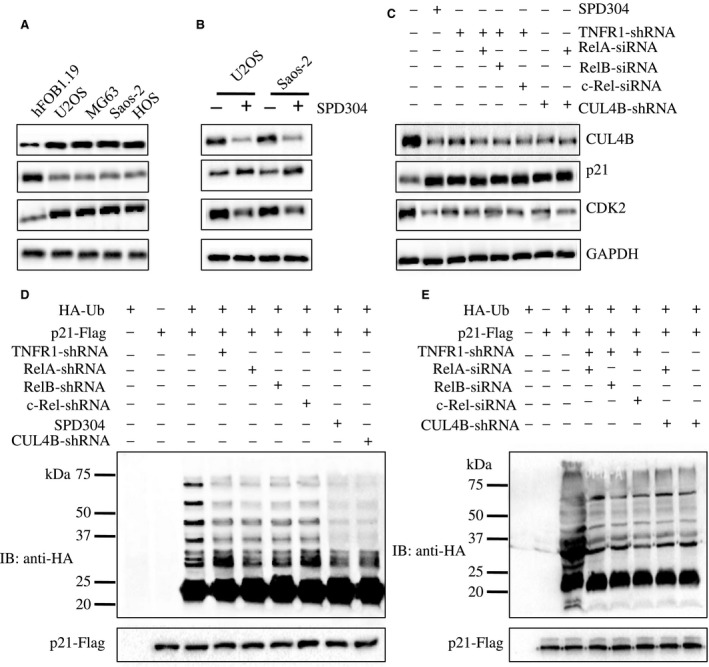
Disruption of the TNF‐α/NF‐κB axis attenuates the ubiquitination of p21. (A) p21 was down‐regulated in osteosarcoma cells. The p21 protein levels in hFOB1.19, U2OS, MG63, Saos‐2, and HOS cells were determined. CUL4B, CDK2, and GAPDH were used as controls. (B) Effects of SPD304 on p21 protein levels. U2OS and Saos‐2 cells were treated with (+) or without (−) SPD304 for 24 h, and the protein levels of CUL4B, p21, CDK2, and GAPDH were determined. (C) Effects of the disruption of TNF‐α/NF‐κB axis on p21 protein levels. U2OS cells treated with SPD304 or U2OS cells transfected with TNFR1‐shRNA, TNFR1‐shRNA + RelA‐siRNA, TNFR1‐shRNA + RelB‐siRNA, TNFR1‐shRNA + c‐Rel‐siRNA, CUL4B‐shRNA, or CUL4B‐shRNA + RelA‐siRNA were assessed for the protein levels of CUL4B, p21, CDK2, and GAPDH. (D–E) Effects of the TNF‐α/NF‐κB axis disruption on p21 ubiquitination. U2OS cells; U2OS cells with knocked down TNFR1, RelA, RelB, c‐Rel, CUL4B, TNFR1 + RelA, TNFR1 + RelB, TNFR1 + c‐Rel, or RelA + CUL4B; and U2OS cells treated with SPD304 were co‐transfected with pCDNA3‐p21‐Flag and HA‐Ubiquitin for 48 h; cells were lysed, immunoprecipitated with Flag antibody, and probed with anti‐HA antibody to detect ubiquitinated p21. The input of p21‐Flag was determined by western blotting using anti‐Flag antibodies.

Our previous results indicated that CUL4B associates with DDB1, RBX1, and DCAF11 to form an E3 ligase, which targets p21^Cip1^ for ubiquitination (Chen *et al*., 2017). In addition, we verified that p21^Cip1^ was down‐regulated, whereas its inhibitory protein CDK2 was up‐regulated in U2OS, MG63, Saos‐2, and HOS cells (Fig. 5A). To further determine whether the inhibition of the TNF‐α/NF‐κB axis affects p21^Cip1^ and CDK2 levels, we treated U2OS and Saos‐2 cells with SPD304 and examined p21^Cip1^ and CDK2 levels. As expected, the inhibition of the TNF‐α/NF‐κB axis increased p21^Cip1^ levels but reduced CDK levels (Fig. 5B). In addition, we knocked down *TNFR1* using shRNA in U2OS cells and obtained the stable U2OS*‐TNFR1‐shRNA* cell line. Using this cell line, we further knocked down *RelA*,* RelB*,* c‐Rel*, and *CUL4B* using corresponding siRNA species, and we examined p21^Cip1^ and CDK2 levels by immunoblot analysis. Our results indicated that p21^Cip1^ levels were significantly increased, whereas CDK2 levels were dramatically down‐regulated after the *TNFR1* down‐regulation or after combined *TNFR1 *+* RelA*,* TNFR1 *+* RelB*,* TNFR1 *+* c‐Rel*, or *TNFR1 *+* CUL4B* down‐regulation (Fig. 5C). However, we did not observe any obvious differences between the single and combined down‐regulation of these genes (Fig. 5C).

The knockdown of any component of the CRL4B^DCAF11^ complex, including *CUL4B*,* DDB1*, or *DCAF11*, attenuated the ubiquitination of p21^Cip1^ (Chen *et al*., 2017). Thus, it is highly likely that inhibition of the TNF‐α/NF‐κB axis decreases CRL4B E3 ligase activity and therefore attenuates p21^Cip1^ ubiquitination levels and ultimately induces p21. To verify this hypothesis, we transfected *pCDNA3‐p21‐Flag* vector alone or co‐transfected the *pCDNA3‐p21‐Flag* vector with HA‐Ub into U2OS*‐TNFR1‐shRNA*, U2OS*‐RelA‐shRNA*, U2OS*‐RelB‐shRNA*, U2OS*‐c‐Rel‐shRNA*, U2OS*‐CUL4B‐shRNA*, and SPD304‐treated U2OS cells before performing *in vivo* ubiquitination analysis. Our results indicated that knocking down *TNFR1*,* RelA*,* RelB*,* c‐Rel*, and *CUL4B* significantly reduced p21^Cip1^ ubiquitination levels (Fig. 5D). However, no obvious differences were observed between the ubiquitination patterns in these cell lines. To further examine the effects of the down‐regulation of *TNFR1*,* RelA*,* RelB*,* c‐Rel*, and *CUL4B* on p21^Cip1^ ubiquitination, we knocked down *RelA*,* RelB*, and *c‐Rel* with their specific siRNA species in the U2OS*‐TNFR1‐shRNA* cell line, and knocked down *RelA* in the U2OS*‐CUL4B‐shRNA* cell line and then co‐transfected the *pCDNA3‐p21‐Flag* vector and HA‐Ub. Our *in vivo* ubiquitination analysis results indicated that no further decrease of p21^Cip1^ ubiquitination level was seen in the cells that underwent dual knockdown compared to those cells with single gene down‐regulation (Fig. 5E). These results were consistent with p21^Cip1^ and CDK2 protein level changes after single or double gene down‐regulation (Fig. 5C), suggesting that the functionality of these genes involves the same pathway.

### 3.7. Inhibition of the TNF‐α/NF‐κB/CUL4B axis inhibits osteosarcoma cell growth

**Figure 6:**
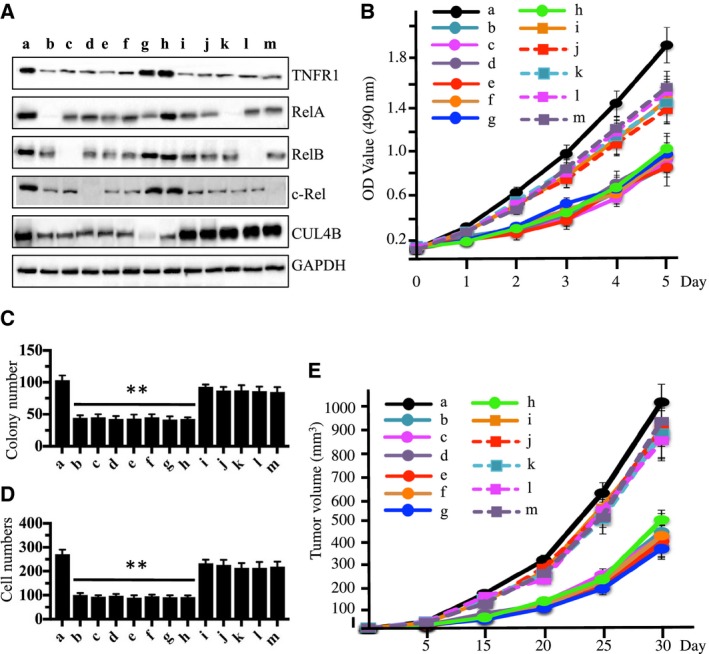
Blockage of TNF‐α/NF‐κB axis inhibits osteosarcoma cell growth. (A) Blockage of the TNF‐α/NF‐κB axis decreased TNFR1, RelA, RelB, c‐Rel, and CUL4B levels. U2OS cells transfected with con‐shRNA (a), U2OS‐TNFR1‐KD + RelA‐KD (b), U2OS‐TNFR1‐KD + RelB‐KD (c), U2OS‐TNFR1‐KD + c‐RelA‐KD (d), U2OS‐SPD304 (e), U2OS‐TNFR1‐KD (f), U2OS‐RelA‐KD + CUL4B‐KD (g), U2OS‐CUL4B‐KD (h), U2OS‐TNFR1‐KD + CUL4B (i), U2OS‐SPD304 + CUL4B (j), U2OS‐TNFR1‐KD + RelA‐KD + CUL4B (k), U2OS‐TNFR1‐KD + RelB‐KD + CUL4B (l), and U2OS‐TNFR1‐KD + c‐Rel‐KD + CUL4B (m) were subjected to western blots to examine the protein levels of TNFR1, RelA, RelB, c‐Rel, and CUL4B. GAPDH was used as a loading control. (B) Blockage of the TNF‐α/NF‐κB axis inhibited osteosarcoma cell proliferation. Cells used in (A) were analyzed using the MTT assay to evaluate cell proliferation with absorbance measurement at 490 nm. (C) Blockage of the TNF‐α/NF‐κB axis decreased colony formation rates. Cells from A were seeded onto 12‐well plates and cultured with 0.1 mL of fresh medium containing 0.5% FBS for two weeks. **P <* *0.001. (D) Blockage of the TNF‐α/NF‐κB axis decreased cell invasion. Cells from A were subjected to Boyden chamber assays, and the invasive cells were counted. **P <* *0.001. (E) Blockage of the TNF‐α/NF‐κB axis decreased the *in vivo* tumor forming ability. Cells from A were injected intradermally into the flanks of mice. Tumor volumes were measured with fine calipers at 5‐day intervals.

Given that knocking down *RelA*,* RelB*, and *c‐Rel* significantly inhibited the proliferation, invasion, colony forming ability, and *in vivo* tumor forming ability of osteosarcoma cells, we sought to determine whether the inhibition of TNF‐α‐mediated signaling has similar effects on osteosarcoma cell growth. U2OS‐*control‐shRNA*, U2OS*‐TNFR1‐shRNA* (TNFR1‐KD), U2OS*‐TNFR1‐shRNA *+ *RelA‐siRNA* (TNFR1‐KD + RelA‐KD), U2OS*‐TNFR1‐shRNA *+ *RelB‐siRNA* (TNFR1‐KD + RelB‐KD), U2OS*‐TNFR1‐shRNA *+ c‐*Rel‐siRNA* (TNFR1‐KD + c‐Rel‐KD), U2OS*‐CUL4B‐shRNA* (CUL4B‐KD), U2OS*‐CUL4B‐shRNA +RelA‐siRNA* (CUL4B‐KD + RelA‐KD), and U2OS‐*control‐shRNA* treated with SPD304 (SPD304) cells were first subjected to immunoblots to examine the protein levels of TNFR1, RelA, RelB, c‐Rel, and CUL4B (Fig. 6A). After confirming protein levels, these cells were subjected to proliferation assays. As shown in Fig. 6B, inhibiting the TNF‐α/NF‐κB/CUL4B axis significantly inhibited osteosarcoma cell proliferation compared with control cells. Among the cells that underwent single and double gene knockdowns as well as SPD304‐treated cells, we did not find obvious differences in proliferation. In addition, these cells were subjected to colony formation and invasion assays. Our results indicated that disrupting the TNF‐α/NF‐κB/CUL4B axis significantly decreased colony forming ability (Figs 6C, S9A) and inhibited cell invasiveness (Figs 6D, S9B). In addition, to evaluate whether knocking down *TNFR1* alone, or its combination with *RelA*,* RelB*, or *c‐Rel*, as well as knocking down *CUL4B *+ *RelA*, would result in the same suppressive effects *in vivo*, we injected nude mice with U2OS‐*control‐shRNA*, U2OS*‐*TNFR1‐KD, U2OS*‐*TNFR1‐KD + RelA‐KD, U2OS*‐*TNFR1‐KD + RelB‐KD, U2OS*‐*TNFR1‐KD + c‐Rel‐KD, U2OS*‐*CUL4B‐KD, U2OS*‐*CUL4B‐KD + RelA‐KD, and U2OS‐SPD304 cells and monitored tumorigenesis for 30 days by measuring tumor volumes at 5‐day intervals. Compared to mice injected with U2OS‐*control‐shRNA*, mice injected with other cells showed significantly slower tumor growth and decreased tumor volumes (53% reduction by day 25) (Fig. 6E). No differences in tumor formation ability or tumor volumes were found among these cells. Similarly, we also overexpressed *CUL4B* in U2OS‐SPD304, U2OS‐TNFR1‐KD, U2OS‐TNFR1‐KD + RelA‐KD, U2OS‐TNFR1‐KD + RelB‐KD, and U2OS‐TNFR1‐KD + c‐Rel‐KD cells to determine whether CUL4B could reverse or partially rescue the decreased cell proliferation, colony formation ability, invasion, and *in vivo* tumor formation ability. Interestingly, overexpressing *CUL4B* significantly rescued these defects (Fig. 6A–E).

Given that the TNF‐α/NF‐κB axis was activated in osteosarcoma cells, we next sought to investigate whether the overexpression of NF‐κB subunits in hFOB1.19 cells would cause CUL4B up‐regulation and p21 down‐regulation similar to that of osteosarcoma cells. Accordingly, we constructed pCDNA3‐RelA‐Flag, pCDNA3‐RelB‐Flag, pCDNA3‐c‐Rel‐Flag, pCDNA3‐p50‐Flag, pCDNA3‐p52‐Flag, and pCDNA3‐CUL4B‐Flag and transfected them into hFOB1.19 cells. As shown in Fig. S10A, overexpressing *RelA*,* RelB*,* c‐Rel*, or *CUL4B* can result in p21 down‐regulation but not p50 or p52 overexpression. Moreover, the overexpression of RelA, RelB, or c‐Rel can up‐regulate CUL4B expression. Consistent with the phenotypes of osteosarcoma cells, the up‐regulation of RelA, RelB, c‐Rel, and CUL4B in hFOB1.19 cells also led to increased colony formation (Fig. S10B) and increased invasion (Fig. S10C). These results, together with the results that knocking down RelA, RelB, c‐Rel, and CUL4B lead to increased p21 levels (Fig. 5C), suggested that the TNF‐α/NF‐κB/CUL4B axis is sufficient and necessary for p21‐dependent cell cycle regulation.

### 3.8. Blocking the TNF‐α/NF‐κB/CUL4B axis induces cell cycle arrest at the S phase

**Figure 7:**
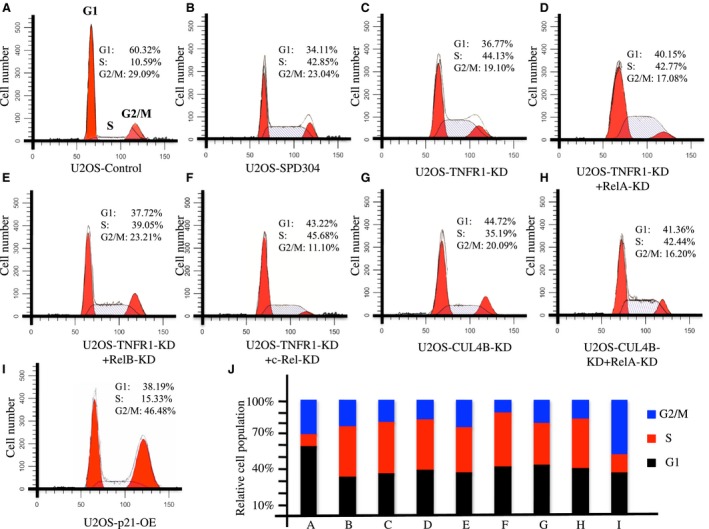
Disruption of the TNF‐α/NF‐κB axis causes cell cycle arrest at the S phase. U2OS cells transfected with Con‐shRNA (Control) (A), U2OS‐SPD304 (B), U2OS‐TNFR1‐KD **(**C**)**, U2OS‐RelA‐KD + RelA‐KD (D), U2OS‐TNFR1‐KD + RelB‐KD (E), U2OS‐TNFR1‐KD + c‐Rel‐KD (F), U2OS‐CUL4B‐KD (G), U2OS‐RelA‐KD + CUL4B‐KD (H), or U2OS‐p21‐OE (I) were subjected to flow cytometry analysis to determine cell cycle distribution. The cell cycle distributions from A to I were quantified in (J).

Our results showed that TNF‐α/NF‐κB/CUL4B axis blockage led to an accumulation of p21 and a reduction in CDK2, which are two key regulators of cell cycle progression; therefore, we hypothesized that the blockage of the TNF‐α/NF‐κB/CUL4B axis would affect cell cycle progression. To verify this hypothesis, we subjected U2OS‐*control‐shRNA*, U2OS*‐*TNFR1‐KD, U2OS*‐*TNFR1‐KD + RelA‐KD, U2OS*‐*TNFR1‐KD + RelB‐KD, U2OS*‐*TNFR1‐KD + c‐Rel‐KD, U2OS*‐*CUL4B‐KD, U2OS*‐*CUL4B‐KD + RelA‐KD, U2OS‐SPD304, and U2OS cells overexpressing pCDNA3‐p21‐Flag (U2OS‐p21‐OE) to flow cytometry analyses. As expected, osteosarcoma cells expressing lower levels of *TNFR1*,* TNFR1 *+* RelA*,* TNFR1 *+* RelB*,* TNFR1 + c‐RelA*,* CUL4B*, and *CUL4B + RelA*; SPD304‐treated cells; and p21‐overexpressing cells exhibited dramatically higher percentages of cells in the S phase (Fig. 7A–J). These results suggested that S phase cell cycle arrest might contribute to cell proliferation, colony formation, cell invasion, and tumor formation abilities observed after the inhibition of the TNF‐α/NF‐κB/CUL4B axis.

## 4. Discussion

**Figure 8:**
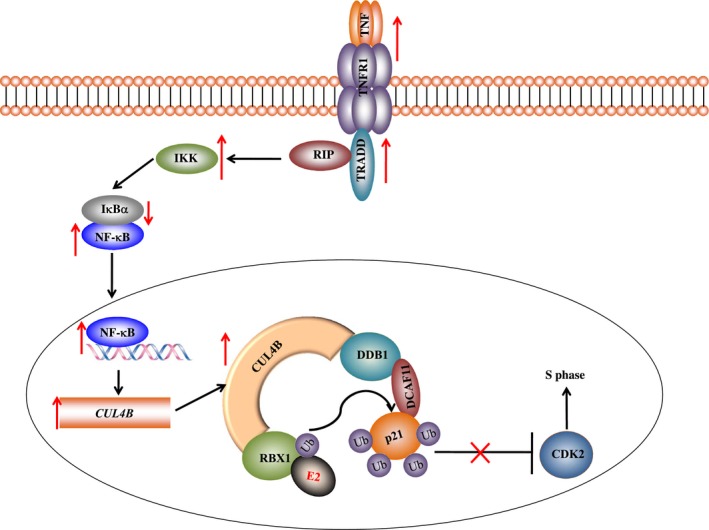
Schematic diagram of the TNF‐α/NF‐κB/CUL4B/p21 axis in human osteosarcoma cells. The activation of TNF‐α signaling facilitates the interaction of TNFR with TRADD, which recruits TRAF2 and RIP. TRAF2 further recruits IKK, enabling its activation by RIP. The activated IKK phosphorylates IκBα and causes the degradation of IκBα, which attenuates its inhibition of NF‐κB and leads to an increase in NF‐κB levels. The accumulated NF‐κB in the cytoplasm then translocates to the nucleus and induces the transcription of *CUL4B*. Overexpressed CUL4B serves as a scaffold that associates with DDB1, RBX1, and DCAF11 to form the CRL4B E3 ligase. The CRL4B E3 ligase promotes ubiquitin transfer from RBX1‐bound E2 to p21 in osteosarcoma cells. The degradation of ubiquitinated p21 abolishes its inhibition of CDK2, thereby disrupting the cell cycle progression.

The CRL4B E3 ligases have been demonstrated to play roles in tumorigenesis (Chen *et al*., 2015, 2017; Hu *et al*., 2012). However, the underlying mechanisms that result in CUL4B overexpression and its upstream signaling remain unclear. In the present study, we discovered that the *CUL4B* promoter region, but not that of other *Cullin* genes, contains an NF‐κB binding site. We revealed that three NF‐κB subunits including RelA, RelB, and c‐Rel, but not p50 and p52, were able to bind to the *CUL4B* promoter, thereby regulating *CUL4B* expression. In addition, we found that the TNF‐α/NF‐κB/CUL4B axis was activated in osteosarcoma cells. Biochemically, the blockage of this pathway resulted in decreased p21 ubiquitination. Physiologically, the inhibition of the TNF‐α/NF‐κB/CUL4B axis significantly reduced proliferation, invasion, and colony formation, induced cell cycle arrest at the S phase, and attenuated the *in vivo* tumor forming ability, and the overexpression of *CUL4B* can greatly reverse these effects. In conclusion, our results provide evidence for a potential pathway through which the activation of the TNF‐α/NF‐κB axis positively regulates CRL4B^DCAF11^ activity, causing p21^Cip1^ degradation and the disruption of cell cycle progression in human osteosarcoma cells (Fig. 8).

Osteosarcoma is a solid bone cancer that is most prevalent in children and young adults. Recently, we found that CUL4B was overexpressed in osteosarcoma and that the activated CUL4B formed an E3 ligase with DDB1, RBX1, and DCAF11, which further ubiquitinated p21 and caused p21 degradation, resulting in the disruption of cell cycle progression (Chen *et al*., 2017). In this study, we used four osteosarcoma cell lines and 54 osteosarcoma patients who were diagnosed at different MSTS stages and confirmed the specificity of *CUL4B* overexpression and its association with osteosarcoma tumorigenesis (Figs S1 and S2). However, due to the limited information for the role of CUL4B in the pathogenesis of cancer including osteosarcoma, we do not know its clinical relevance. Based on our examination results in 54 osteosarcoma patients, it is possible that *CUL4B* levels affect tumor survival. The Cullin family has six classic members and two atypical members, and we determined why only *CUL4B* was overexpressed in osteosarcoma cells. Given that *CUL4B* was induced at the mRNA and protein levels, it is highly possible that *CUL4B* was regulated by a transcription factor in osteosarcoma cells. Therefore, we analyzed the promoter regions of *CUL1*,* CUL2*,* CUL3*,* CUL4A*,* CUL4B*,* CUL5*, and *CUL7* and found that only the promoter region of *CUL4B* but not other *Cullin* genes contained a conserved NF‐κB transcription factor‐binding site (GGGGTTTCCC) (Fig. 1A). In addition, we compared other transcription factor sites, including activator protein 1 (*AP‐1*), *c‐Myc*, p53, and specificity protein 1 (*SP1*), in the promoter regions of *Cullin* genes. We did not find any specificity of these transcription factors in the *CUL4B* promoter region (data not shown). The NF‐κB transcription factor has five subunits, and we tested their abilities to regulate *CUL4B* expression in osteosarcoma cells. Surprisingly, we found that RelA, RelB, and c‐Rel, but not p50 and p21, could activate *CUL4B* expression (Fig. 1C,D). One potential explanation for this effect is that the p50 and p52 proteins have no intrinsic ability to activate gene transcription, and they may act as transcriptional repressors when binding to κB elements as homodimers. In addition, we detected Cullin levels when RelA, RelB, and c‐Rel were down‐regulated and found that the CUL4B protein levels were significantly reduced (Figs S3 and S4). These results demonstrated that *CUL4B* overexpression in osteosarcoma cells is caused by the specific regulation of the three NF‐κB subunits RelA, RelB, and c‐Rel.

NF‐κB can be activated by a variety of stimuli, and the activation of NF‐κB has important roles in the initiation and progression of cancer (Gupta *et al*., 2010; Hoesel and Schmid, 2013). Generally, NF‐κB is thought to be located in the cytoplasm in most healthy cell types, whereas it is translocated to the nucleus by certain stimuli and in most cancer cells (Hoesel and Schmid, 2013). In our experiments, we found that RelA, RelB, p52, and CUL4B were primarily located in the cytoplasm in hFOB1.19 cells and healthy clinical tissues (Figs S5 and S6), whereas RelA, RelB, and CUL4B were much more abundant in U2OS cells and cancerous tissues (Figs S5 and S6). By comparing the localization patterns of RelA, RelB, and CUL4B in our experiments to previously published results by other groups and the datasheet of manufacturers, we found that these proteins were not completely translocated to the nucleus in U2OS cells (Fig. S5). To exclude technical problems in our experiments, we also used a human melanoma cell line A375 as a control to determine the localization patterns of RelA, RelB, and CUL4B. As shown in Fig. S11, we found all these proteins located in the nucleus. Combined with the nuclear localization patterns of RelA, RelB, and CUL4B in a malignant patient (patient 4, MSTS IV), we speculate that RelA, RelB, and CUL4B were not completely located in the nucleus because of the cell type. To further confirm this speculation, we selected RelA as the representative to examine its nuclear localization in the other five cell lines including two pancreatic adenocarcinoma cell lines Panc‐28 and CFPAC‐1, a lung cancer cell line H1299, a breast cancer cell line MCF‐7, and a carcinoma cell line Fadu. These cells exhibited different cell proliferation, RelA levels, and invasion abilities (Fig. S12). As shown in Fig. S13A, the IMF staining results with anti‐RelA antibody indicated that Panc‐28 and H1299 cells had incomplete nuclear translocation, while RelA was completely translocated into the nucleus in the other three cell lines. Moreover, we also examined the localization pattern of RelA in clinical samples from four patients who underwent at MSTS stage I‐IV based on the histopathological features (as shown in Fig. S2C). The IMF results indicated that RelA was dominant in the nucleus in cancerous tissues compared to the noncancerous tissues (Fig. S13B). Interestingly, with the increase in tumor malignancy in osteosarcoma patients, the proportion of nuclear translocation of RelA gradually increased (Fig. S13B). These results clearly demonstrated that NF‐κB translocation might be different in different types of cells.

We found three NF‐κB subunits that can activate *CUL4B* expression. Therefore, we assessed the activation of NF‐κB and its upstream signaling in osteosarcoma cells. As expected, we found the activation of the TNF‐α/NF‐κB axis (Figs 2 and 4), and the knockdown of *TNFR1*,* RelA*,* RelB*,* c‐Rel*, and *CUL4B* showed similar effects on the proliferation, invasion, colony formation, and the *in vivo* tumor forming ability of cells (Figs 3 and 6). Interestingly, no additional severe phenotypes were observed when we simultaneously knocked down two genes in this pathway, such as TNFR1‐KD + RelA‐KD, TNFR1‐KD + RelB‐KD, TNFR1‐KD + c‐Rel‐KD, and CUL4B‐KD + RelA‐KD (Fig. 6). In addition, the overexpression of *CUL4B* in these cells was able to significantly reverse their growth defects (Figs 3 and 6), further suggesting that the TNF‐α/NF‐κB axis mainly contributed to *CUL4B* expression in osteosarcoma cells. These results suggested that these genes function in the same pathway. Importantly, these results provide an opportunity to target the TNF‐α/NF‐κB/CUL4B axis in treating osteosarcoma.

Once we addressed the questions regarding the regulation of *CUL4B* overexpression and its upstream signaling, we next wanted to know whether the disruption of TNF‐α/NF‐κB would affect the downstream targets of CUL4B, including p21^Cip1^, a newly identified substrate of the CRL4B E3 ligase. To this end, we examined the ubiquitination of p21^Cip1^ in cells with knocked down *TNFR1*,* RelA*,* RelB*,* c‐Rel*, or *CUL4B* or the simultaneous knockdown of TNFR1‐KD + RelA‐KD, TNFR1‐KD + RelB‐KD, TNFR1‐KD + c‐Rel‐KD, and CUL4B‐KD + RelA‐KD. Consistent with the physiological phenotypes, knocking down these genes caused similar effects on p21^Cip1^ ubiquitination and cell cycle progression (Figs 5 and 7). These results suggested that TNF‐α, NF‐κB, and CUL4B function in the same pathway and contribute to p21^Cip1^ ubiquitination and osteosarcoma tumorigenesis. Importantly, these results support a new model and a new signaling pathway in which the TNF‐α/NF‐κB axis‐mediated overexpression of *CUL4B* results in the ubiquitination of p21^Cip1^, leading to the attenuation of p21 levels and the accumulation of CDK2.

In summary, our results uncovered two major findings: (1) We demonstrated the underlying mechanisms that are involved in *CUL4B* overexpression in human osteosarcoma cells and that NF‐κB subunits specially bind to its promoter region and positively regulate its expression at the transcriptional level. (2) We revealed the activation of the TNF‐α/NF‐κB axis in human osteosarcoma cells, and the blockage of this pathway significantly inhibited osteosarcoma cell growth *in vitro* and *in vivo*, which may present an efficient approach in osteosarcoma therapy.

## Authors’ contributions

ZC, HS, and CZ designed the research. BC and CZ performed the major portion of the experiments. KJ and LL performed parts of the research. CZ, BC, ZC, and HS analyzed the data, tested statistics, and coordinated the figures. ZC and HS wrote the article. ZC and BC revised the article.

## Supporting information


**Fig. S1.** CUL4B is overexpressed in osteosarcoma cells and cancerous tissues from osteosarcoma patients.

**Fig. S2.** CUL4B is significantly induced in cancerous tissues from osteosarcoma patients.

**Fig. S3.** Knockdown of *RelA*,* RelB*, or *c‐Rel* down‐regulates *CUL4B* mRNA levels.

**Fig. S4.** Knockdown of *RelA*,* RelB*, or *c‐Rel* down‐regulates CUL4B protein levels.

**Fig. S5.** NF‐κB subunits and CUL4B are abundant in the nucleus.

**Fig. S6.** NF‐κB subunits and CUL4B are translocated to the nucleus in malignant samples.

**Fig. S7.** Expression patterns of NF‐κB targets in osteosarcoma cells.

**Fig. S8.** Overexpression of *IL‐6* cannot reverse cell growth defects caused by knocking down RelA, RelB, or c‐Rel.

**Fig. S9.** Disruption of TNF‐α/NF‐κB axis decreases colony formation rates and cell invasion.

**Fig. S10**. Overexpression of *RelA*,* RelB*,* c‐Rel*, or *CUL4B* in hFOB1.19 cells results in effects similar to those in U2OS cells.

**Fig. S11.** NF‐κB subunits and CUL4B were localized in the nucleus in a melanoma cell line.

**Fig. S12.** Cell growth and invasion in different cell types.

**Fig. S13.** Different cancer cell lines exhibited different nuclear levels of RelA.

**Table S1.** siRNA and shRNA information.
**Table S2.** The clinicopathological futures of 54 osteosarcoma patients and miR‐300 expression.
