# Supplementary figures and images for: Activation of TNF‐α/NF‐κB axis enhances CRL4BDCAF 11 E3 ligase activity and regulates cell cycle progression in human osteosarcoma cells
Source: Mol Oncol. 2018 Feb 20;12(4):476–94. doi: 10.1002/1878-0261.12176 (PMC5891038; doi:10.1002/1878-0261.12176)

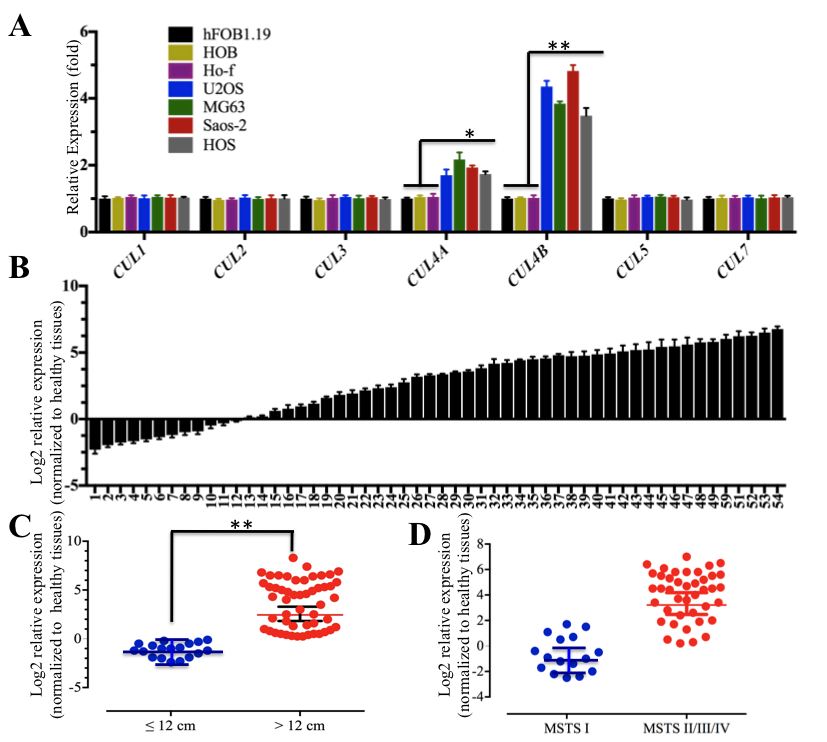

Supplement: Supplementary file 1 — Fig. S1. CUL4B is overexpressed in osteosarcoma cells and cancerous tissues from osteosarcoma patients. [file MOL2-12-476-s001.tif]

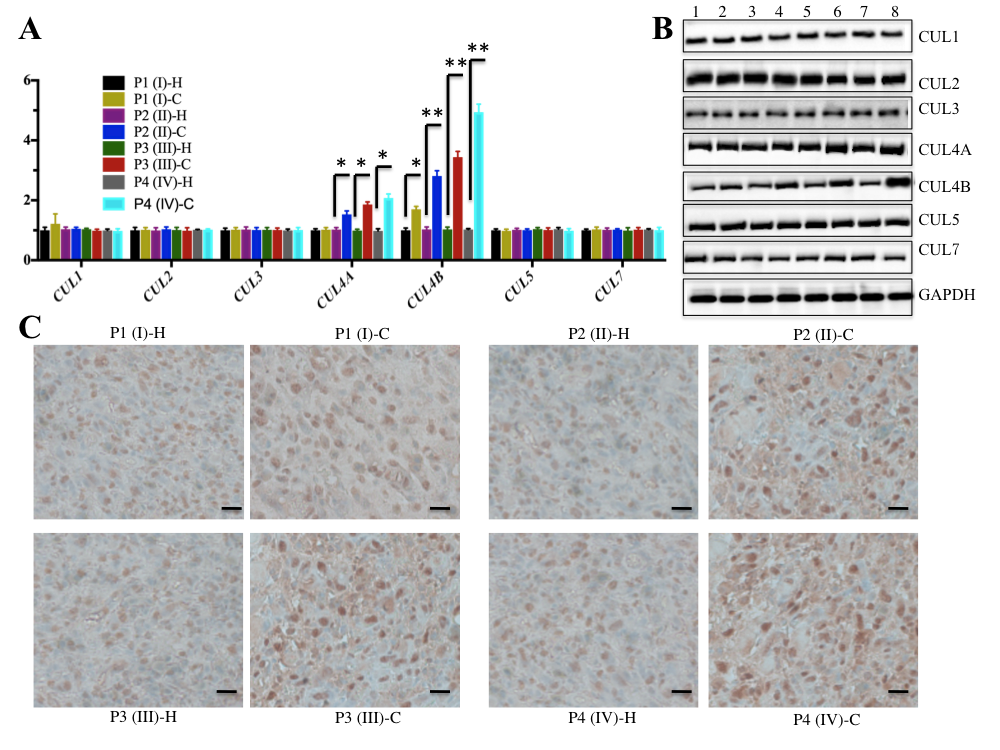

Supplement: Supplementary file 2 — Fig. S2. CUL4B is significantly induced in cancerous tissues from osteosarcoma patients. [file MOL2-12-476-s002.tif]

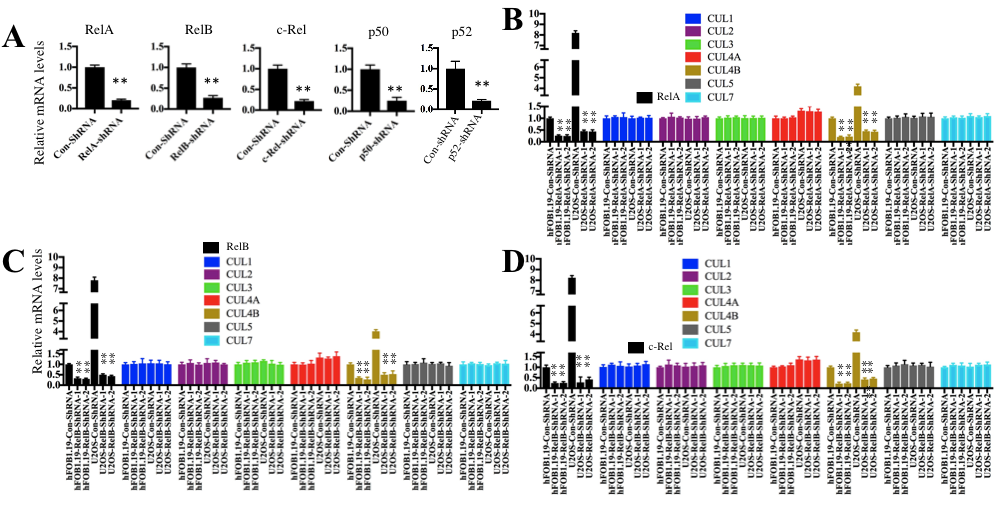

Supplement: Supplementary file 3 — Fig. S3. Knockdown of RelA, RelB, or c‐Rel down‐regulates CUL4B mRNA levels. [file MOL2-12-476-s003.tif]

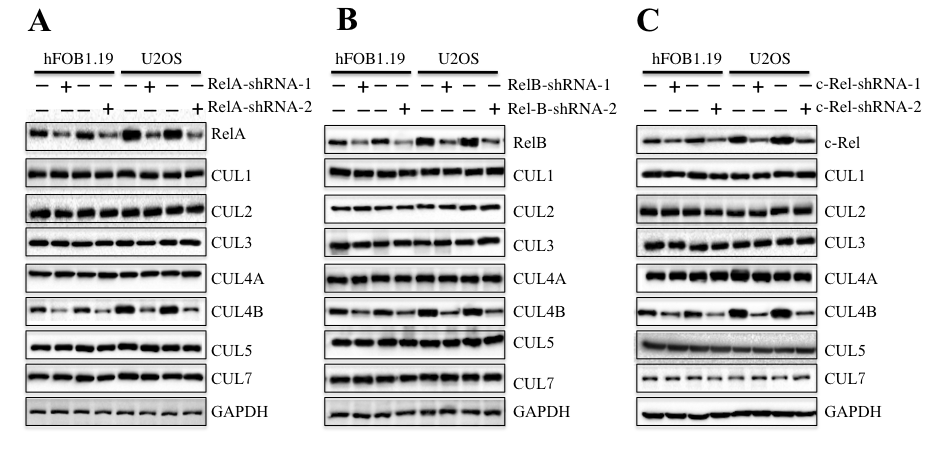

Supplement: Supplementary file 4 — Fig. S4. Knockdown of RelA, RelB, or c‐Rel down‐regulates CUL4B protein levels. [file MOL2-12-476-s004.tif]

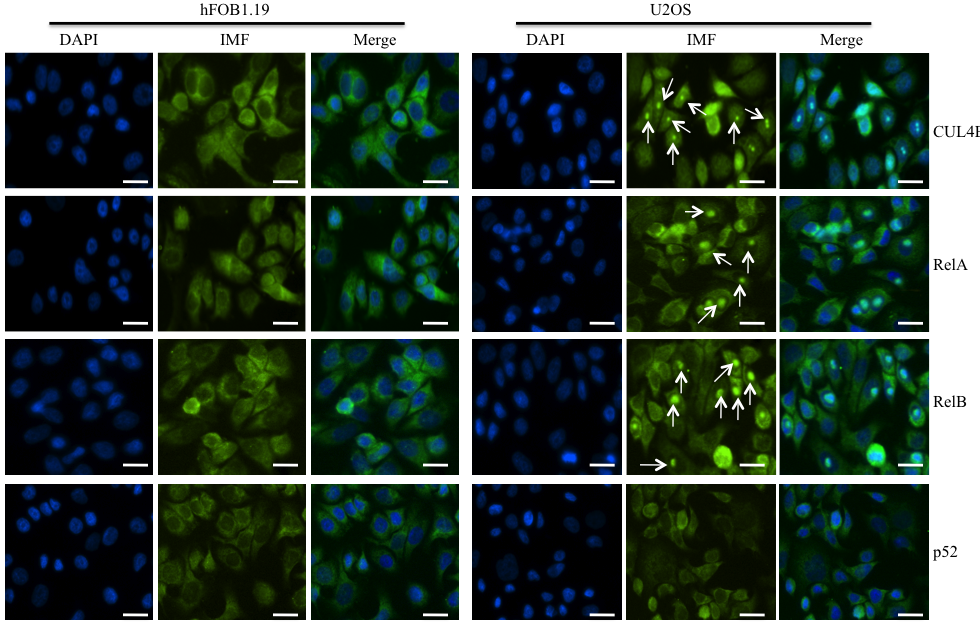

Supplement: Supplementary file 5 — Fig. S5. NF‐κB subunits and CUL4B are abundant in the nucleus. [file MOL2-12-476-s005.tif]

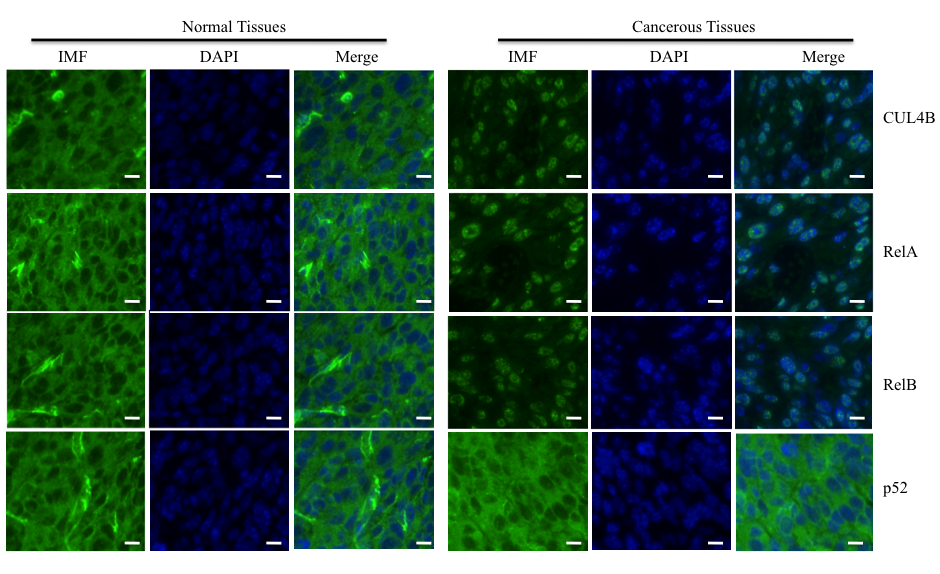

Supplement: Supplementary file 6 — Fig. S6. NF‐κB subunits and CUL4B are translocated to the nucleus in malignant samples. [file MOL2-12-476-s006.tif]

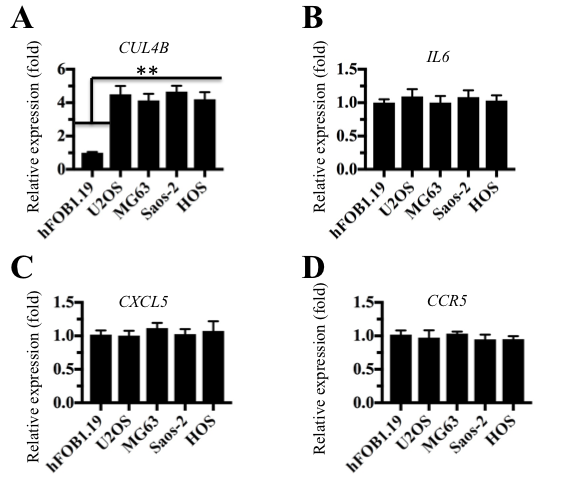

Supplement: Supplementary file 7 — Fig. S7. Expression patterns of NF‐κB targets in osteosarcoma cells. [file MOL2-12-476-s007.tif]

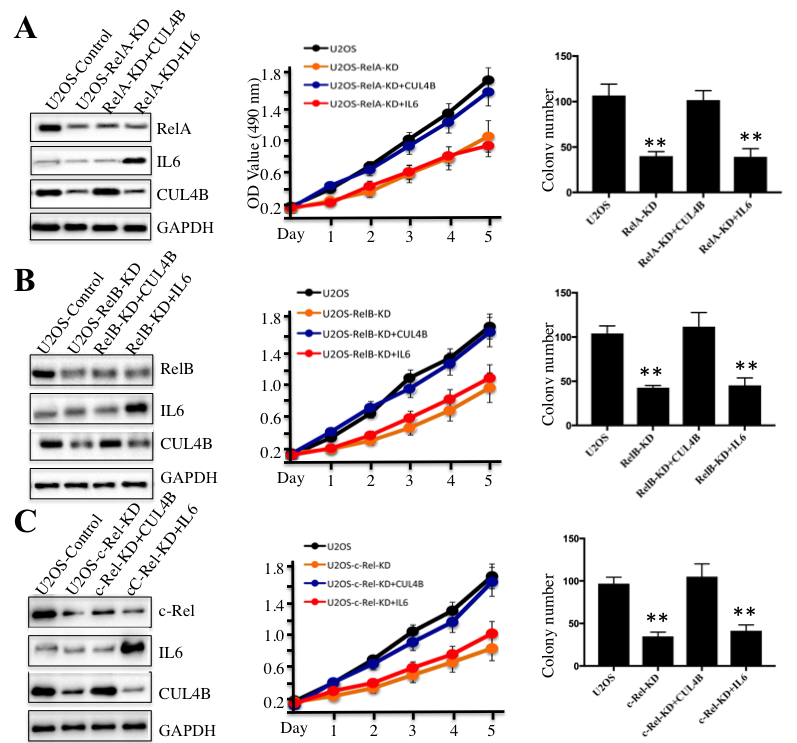

Supplement: Supplementary file 8 — Fig. S8. Overexpression of IL‐6 cannot reverse cell growth defects caused by knocking down RelA, RelB, or c‐Rel. [file MOL2-12-476-s008.tif]

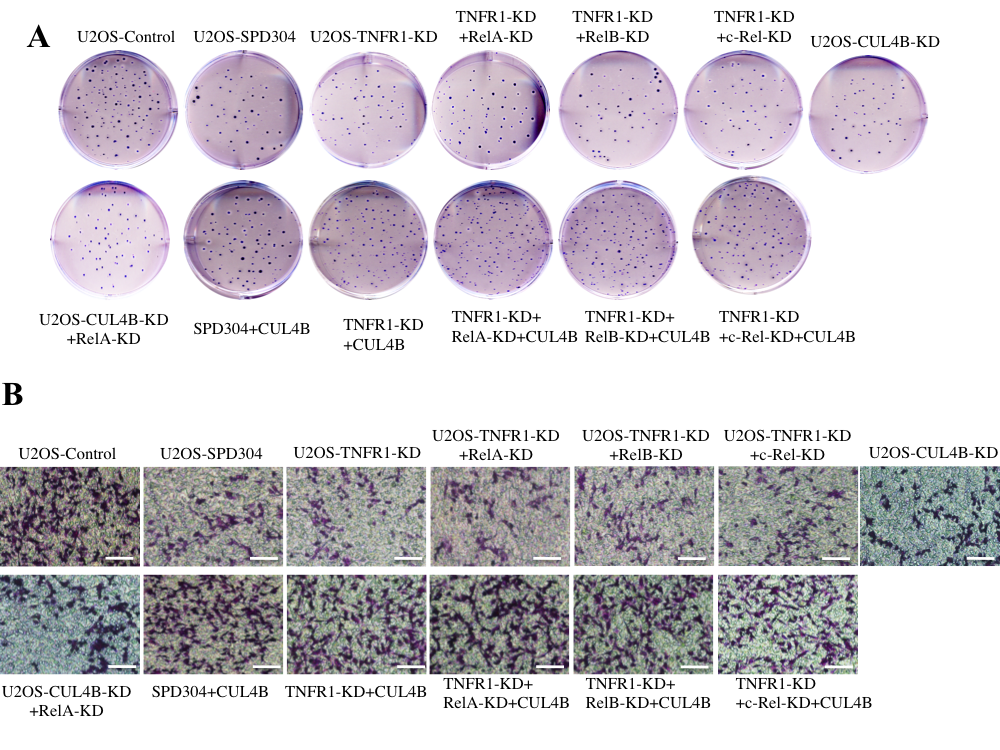

Supplement: Supplementary file 9 — Fig. S9. Disruption of TNF‐α/NF‐κB axis decreases colony formation rates and cell invasion. [file MOL2-12-476-s009.tif]

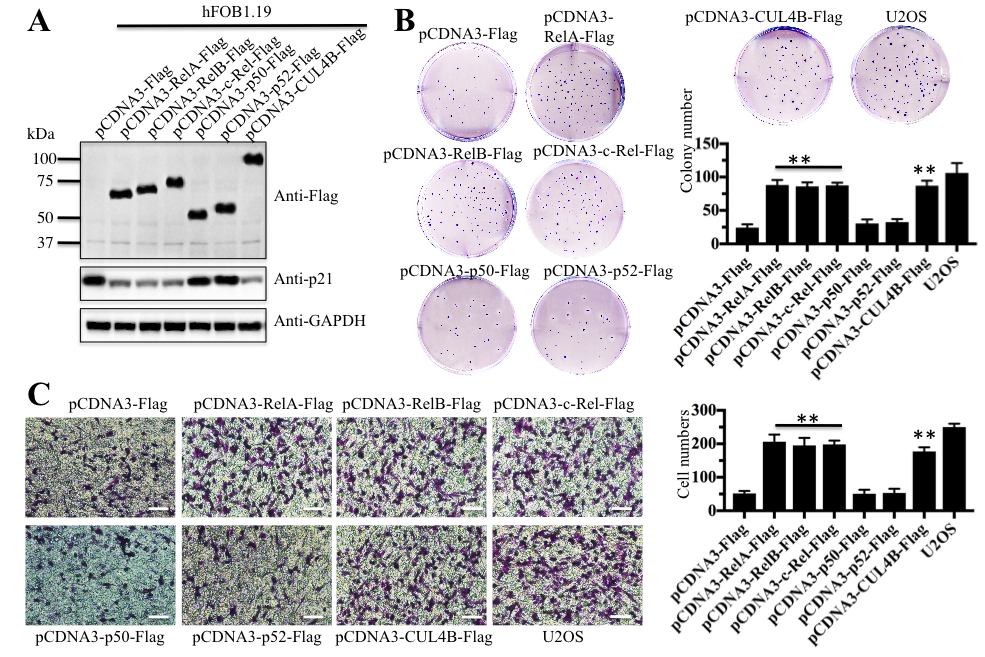

Supplement: Supplementary file 10 — Fig. S10. Overexpression of RelA, RelB, c‐Rel, or CUL4B in hFOB1.19 cells results in effects similar to those in U2OS cells. [file MOL2-12-476-s010.tif]

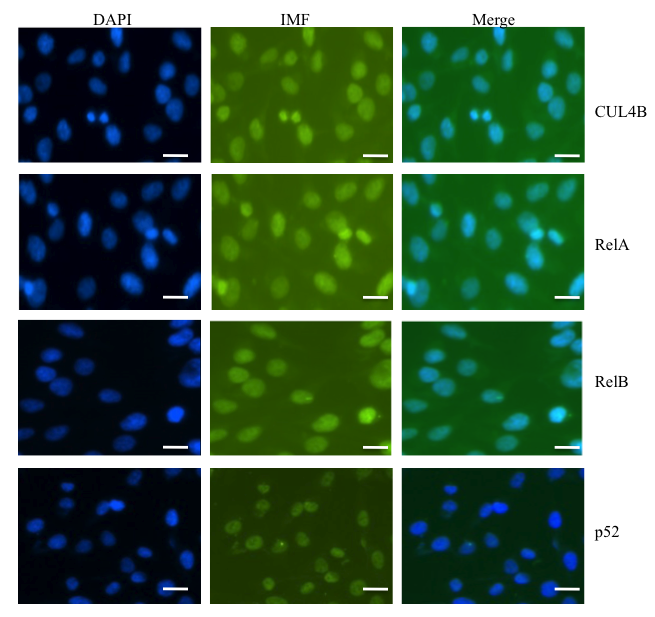

Supplement: Supplementary file 11 — Fig. S11. NF‐κB subunits and CUL4B were localized in the nucleus in a melanoma cell line. [file MOL2-12-476-s011.tif]

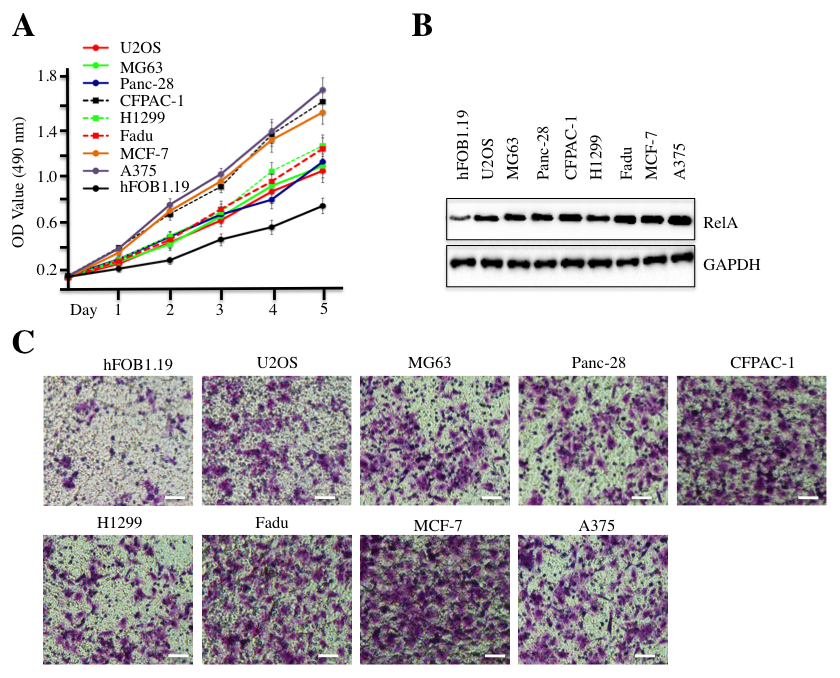

Supplement: Supplementary file 12 — Fig. S12. Cell growth and invasion in different cell types. [file MOL2-12-476-s012.tif]

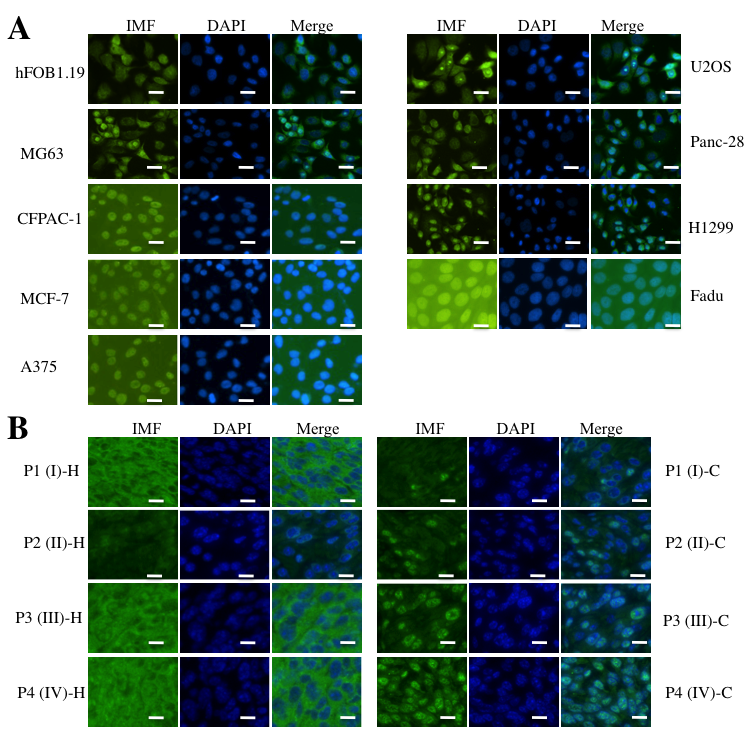

Supplement: Supplementary file 13 — Fig. S13. Different cancer cell lines exhibited different nuclear levels of RelA. [file MOL2-12-476-s013.tif]
