# Supplementary material for: Activation of TNF‐α/NF‐κB axis enhances CRL4BDCAF 11 E3 ligase activity and regulates cell cycle progression in human osteosarcoma cells
Source: Mol Oncol. 2018 Feb 20;12(4):476–94. doi: 10.1002/1878-0261.12176 (PMC5891038; doi:10.1002/1878-0261.12176)
Supplement: Supplementary file 14 — Table S1. siRNA and shRNA information. Table S2. The clinicopathological futures of 54 osteosarcoma patients and miR‐300 expression. [file MOL2-12-476-s014.docx]

**
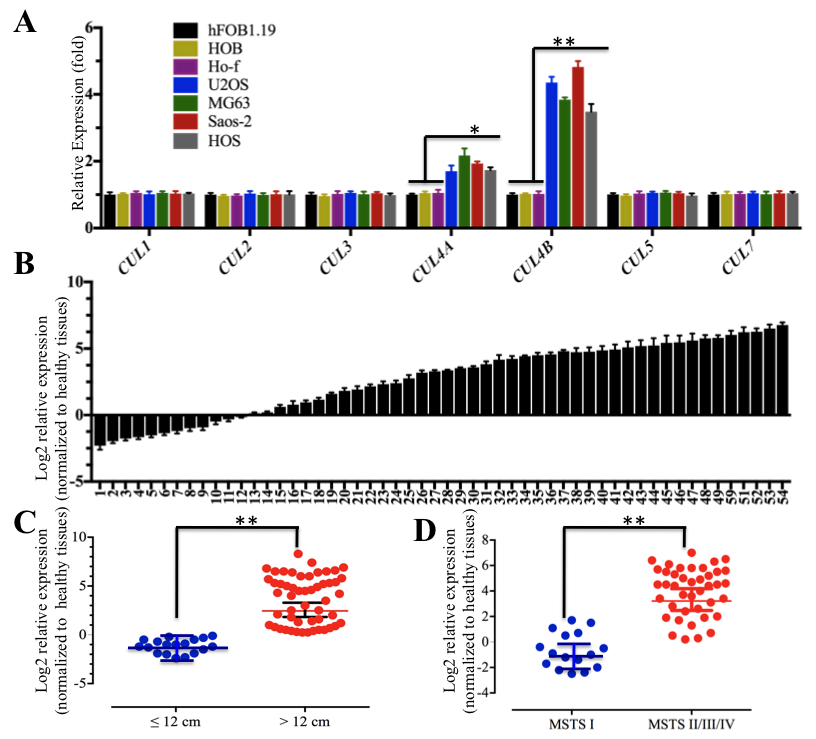
**

**Supplementary Figure 1. CUL4B is overexpressed in osteosarcoma cells and cancerous tissues from osteosarcoma patients.**

1. *Cullin* mRNA levels, including *CUL1*, *CUL2*, *CUL3*, *CUL4A*, *CUL4B*, *CUL5*, and *CUL7*, were determined by qRT-PCR in hFOB1.19, HOB, Ho-f, U2OS, MG63, Saos-2, and HOS cells. Expression levels were normalized to β-Actin in each cell line, and the resulting ratios to hFOB1.19 cells were arbitrarily defined as 1-fold. **(B)** Expression of *CUL4B* in osteosarcoma cancerous tissues is shown. Relative expression of *CUL4B* in osteosarcoma tumors (n = 54) was normalized to corresponding adjacent normal tissues (n = 54), ***P* < 0.001. **(C** and **D)** The expression of *CUL4B* was positively correlated with osteosarcoma tumor size and MSTS stage. The expression of *CUL4B* was significantly higher in larger tumors (tumor maximal diameter ≥ 12 cm) **(C)** and was significantly higher in osteosarcoma patients with advanced MSTS stages (II/III/IV) compared with an earlier MSTS stage (I) **(D)**. ** *P* < 0.001.


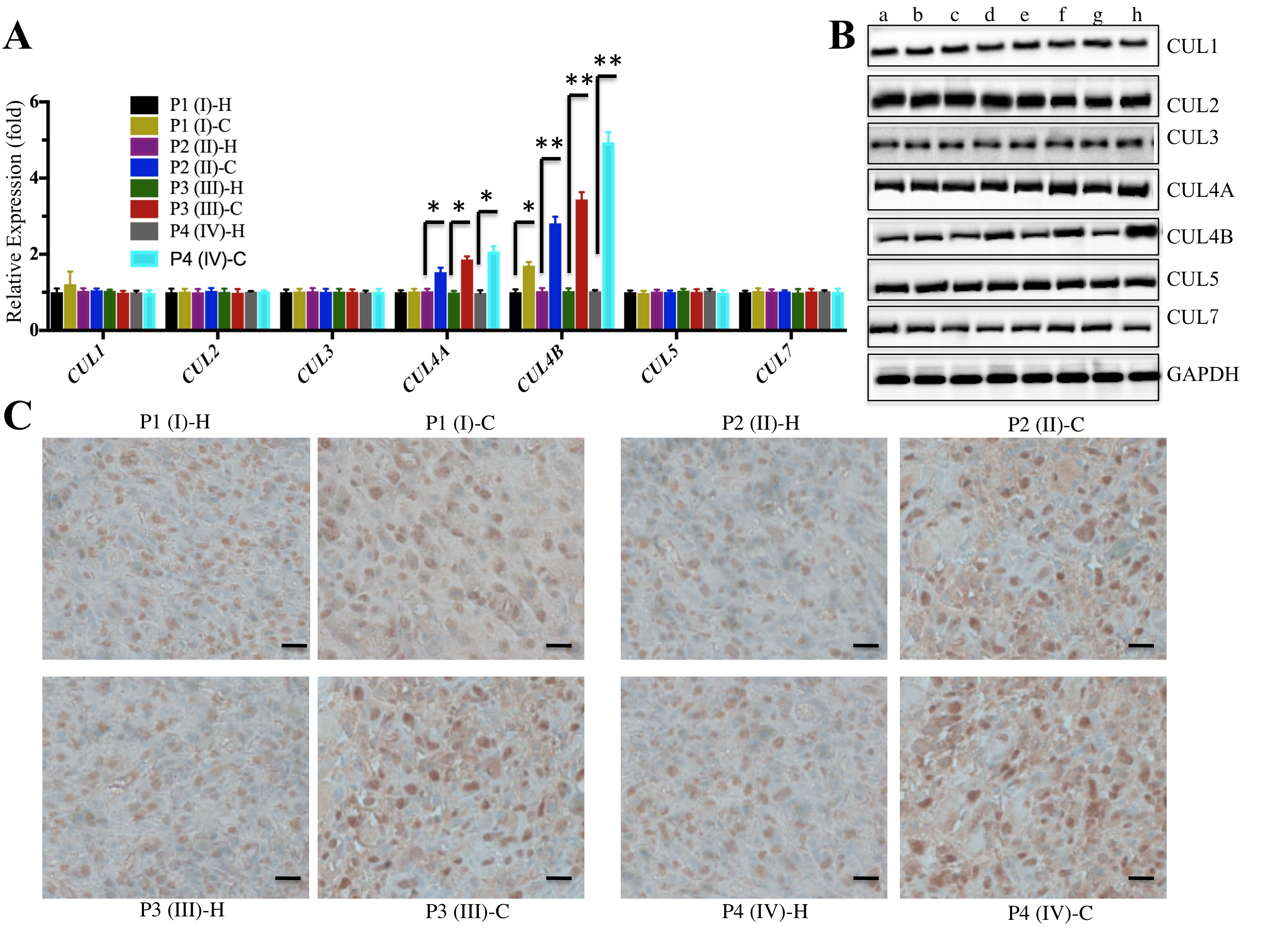


**Supplementary Figure 2. CUL4B is significantly induced in cancerous tissues from osteosarcoma patients.**

**(A)** *Cullin* mRNA levels, including *CUL1*, *CUL2*, *CUL3*, *CUL4A*, *CUL4B*, *CUL5*, and *CUL7*, were determined by qRT-PCR in cancerous tissues obtained from four osteosarcoma patients at different MSTS stages (P1, patient 1, who was MSTS stage I; H, healthy tissue; C, cancerous tissue). Expression levels were normalized to β-Actin levels in each tissue, and the resulting ratios to healthy tissues were arbitrarily defined as 1-fold. Representative data from three independent experiments are shown. **P*<0.05 and ***P*<0.001. **(B)** Cullin protein levels, including CUL1, CUL2, CUL3, CUL4A, CUL4B, CUL5, and CUL7, were determined by Western blotting with cancerous tissues obtained from four osteosarcoma patients at different MSTS stages (a, c, e, and g are the healthy tissues of patients 1, 2, 3, and 4, respectively; b, d, f, and h are the cancerous tissues of patient 1, 2, 3, and 4, respectively). GAPDH was used as a loading control. **(C)** CUL4B was up-regulated in cancerous tissues from four osteosarcoma patients. IHC staining was performed to detect CUL4B using its specific antibody. Bars=100 μm.


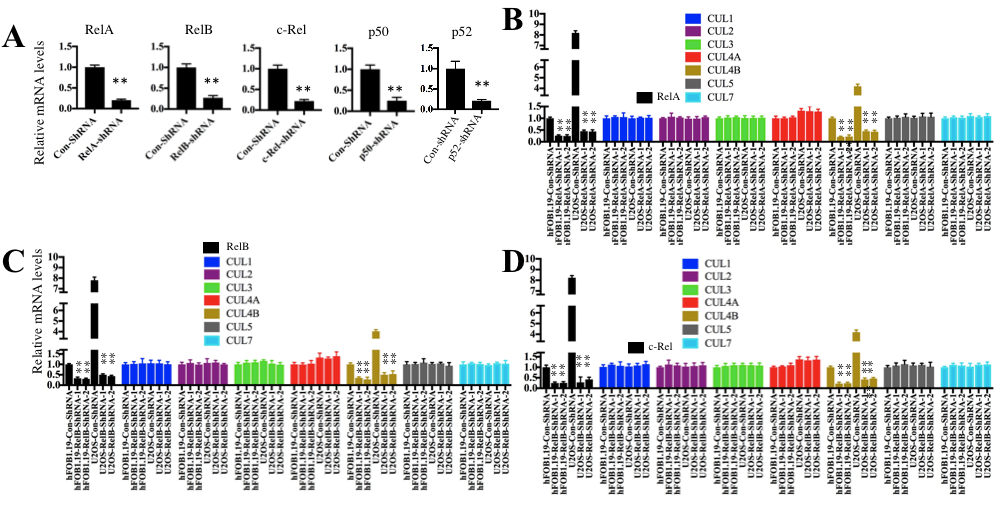


**Supplementary Figure 3. Knockdown of *RelA*, *RelB*, or *c-Rel* down-regulates *CUL4B* mRNA levels.**

**(A)** The knock down efficiency of *RelA*, *RelB*, *c-Rel*, *p50*, and *p52* in U2OS cells for ChIP assays. U2OS cells were transfected with shRNAs against NF-κB subunits to down-regulate *RelA*, *RelB*, *c-Rel*, *p50*, and *p52*. Expression levels of *RelA*, *RelB*, *c-Rel*, *p50*, and *p52* were normalized to β-Actin in each cell line, and the resulting ratios to U2OS cells expressing con-shRNA were arbitrarily defined as 1-fold. **(B-D)** The hFOB1.19 and U2OS cells with down-regulated *RelA* **(B)**, *RelB* **(C)**, or *c-Rel* **(D)** were subjected to qRT-PCR to determine the mRNA levels of *Cullins*. Expression levels of *CUL1*, *CUL2*, *CUL3*, *CUL4A*, *CUL4B*, *CUL5*, and *CUL7* were determined by qRT-PCR with normalization to β-Actin in each cell line, and the resulting ratios to hFOB1.19 cells expressing con-shRNA were arbitrarily defined as 1-fold. ***P*<0.001

**
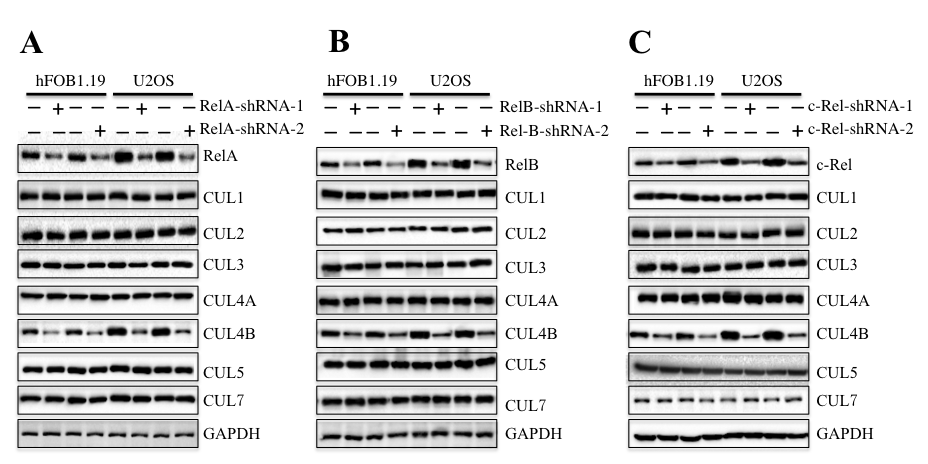
**

**Supplementary Figure 4. Knockdown of *RelA*, *RelB*, or *c-Rel* down-regulates CUL4B protein levels.**

The hFOB1.19 and U2OS cells with down-regulated *RelA* **(A)**, *RelB* **(B)**, or *c-Rel* **(C)** accomplished with two different corresponding shRNAs (shRNA-1 and -2) each were subjected to immunoblots to determine the protein levels of Cullins using appropriate antibodies.


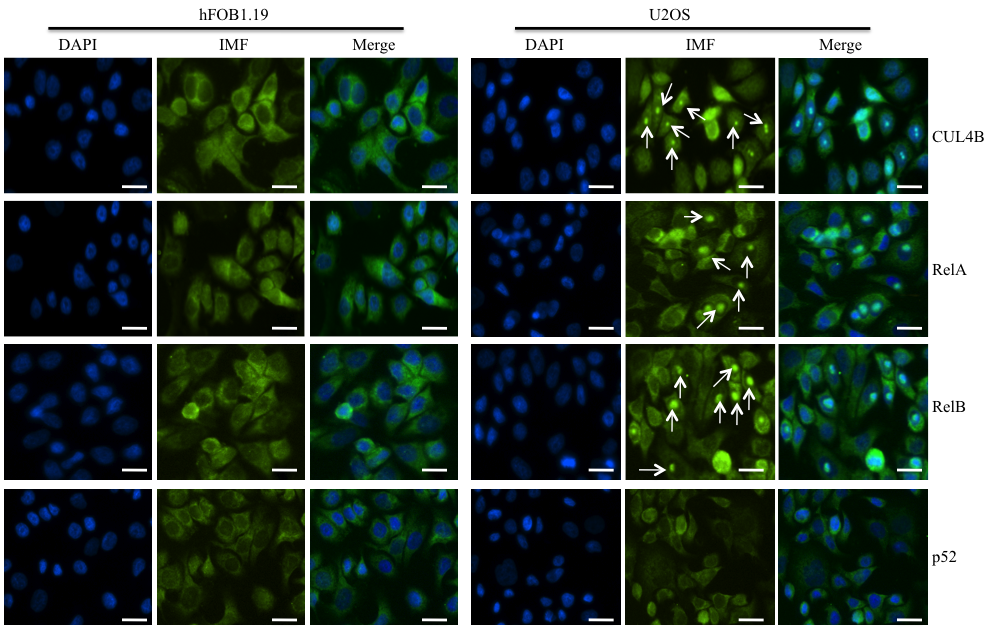


**Supplementary Figure 5. NF-κB subunits and CUL4B are abundant in the nucleus.**

The hFOB1.19 and U2OS cells were subjected to immunofluorescence (IMF) staining with anti-CUL4B, anti-RelA, anti-RelB, and anti-p52 antibodies. The nuclei are counterstained with DAPI (blue, left panel). The localization and levels of CUL4B, RelA, RelB, and p52 protein were shown as green (middle panel), and the merged images were in the right panel. Bars = 25 µm.

**
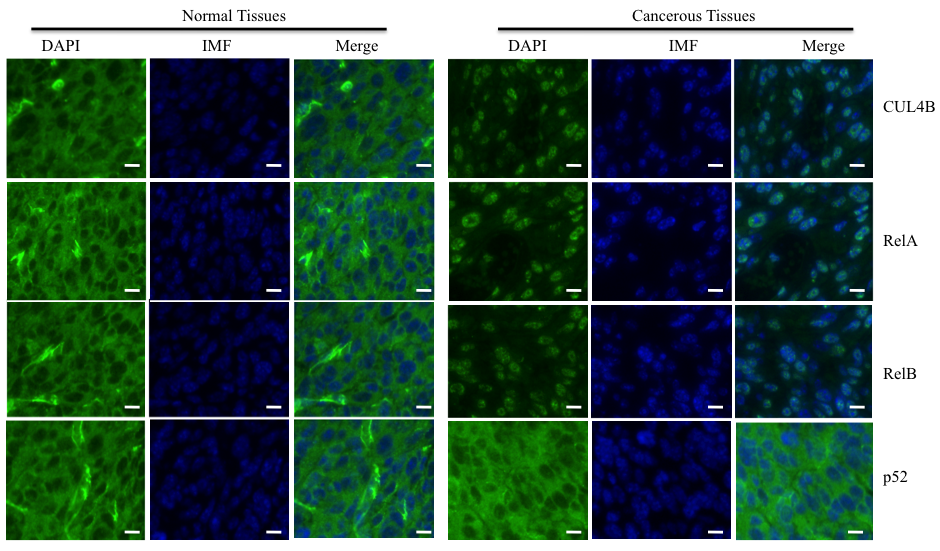
**

**Supplementary Figure 6. NF-κB subunits and CUL4B are translocated to the nucleus in malignant samples.**

The non-cancerous and cancerous samples from patient 4, MSTS IV, were subjected to immunofluorescence (IMF) staining with anti-CUL4B, anti-RelA, anti-RelB, and anti-p52 antibodies. The nuclei are counterstained with DAPI (blue, left panel). The localization and levels of CUL4B, RelA, RelB, and p52 protein are shown as green (middle panel), and the merged images are in the right panel. Bars = 25 µm.

**
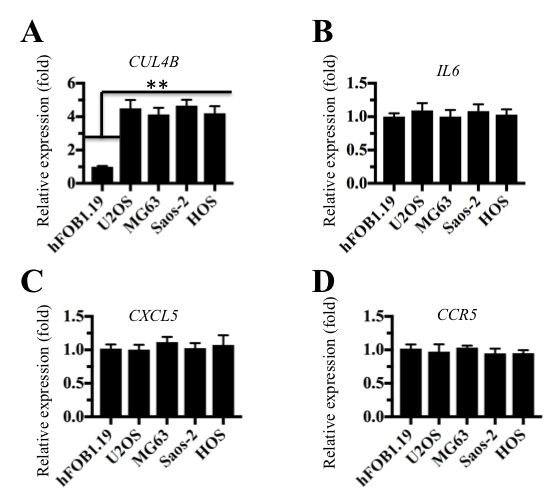
**

**Supplementary Figure 7. Expression patterns of NF-κB targets in osteosarcoma cells.**

The expression of four representative NF-κB targets, including *CUL4B* **(A)**, *IL6* **(B)**, *CXCL5* **(C)**, and *CCR5* **(D)**, was determined by qRT-PCR in hFOB1.19, U2OS, MG63, Saos-2, and HOS cells. Expression levels were normalized to β-Actin in each cell line, and the resulting ratios to hFOB1.19 cells were arbitrarily defined as 1-fold.

**
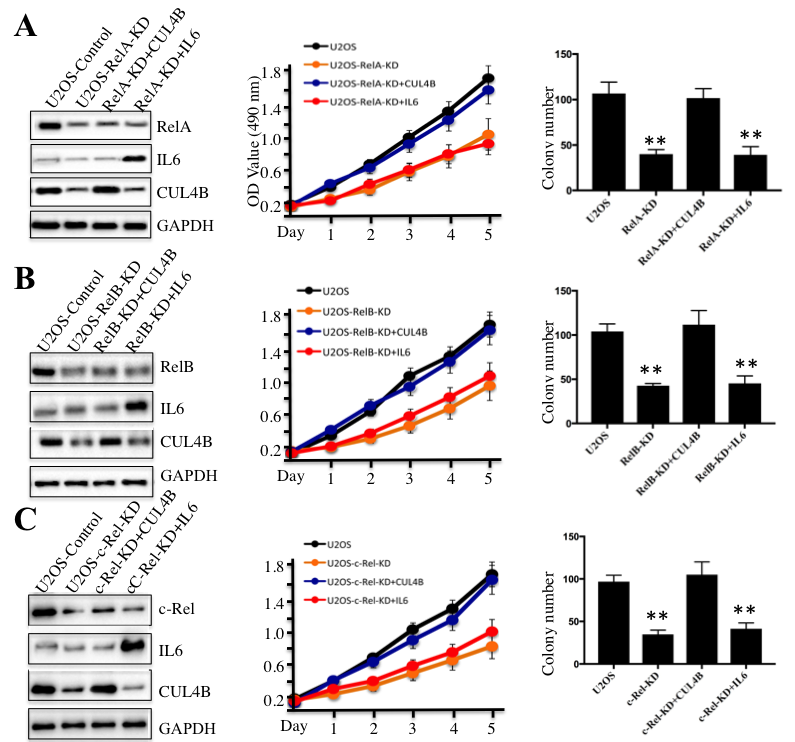
**

**Supplementary Figure 8. Overexpression of *IL-6* cannot reverse cell growth defects caused by knocking down RelA, RelB, or c-Rel.**

U2OS cells transfected with Con-shRNA (U2OS-Control), RelA-shRNA (U2OS-RelA-KD) **(A)**, RelB-shRNA (U2OS-RelB-KD) **(B)**, or c-Rel-shRNA (U2OS-c-Rel-KD) **(C)** were transfected with CUL4B or IL-6 overexpression plasmids. The protein levels of NF-κB subunits, IL-6, CUL4B, and GAPDH were measured by Western blots (left panels), and MTT assays were performed to evaluate cell proliferation with absorbance measured at 490 nm (middle panels) and colony formation (right panels). ***P*<0.001


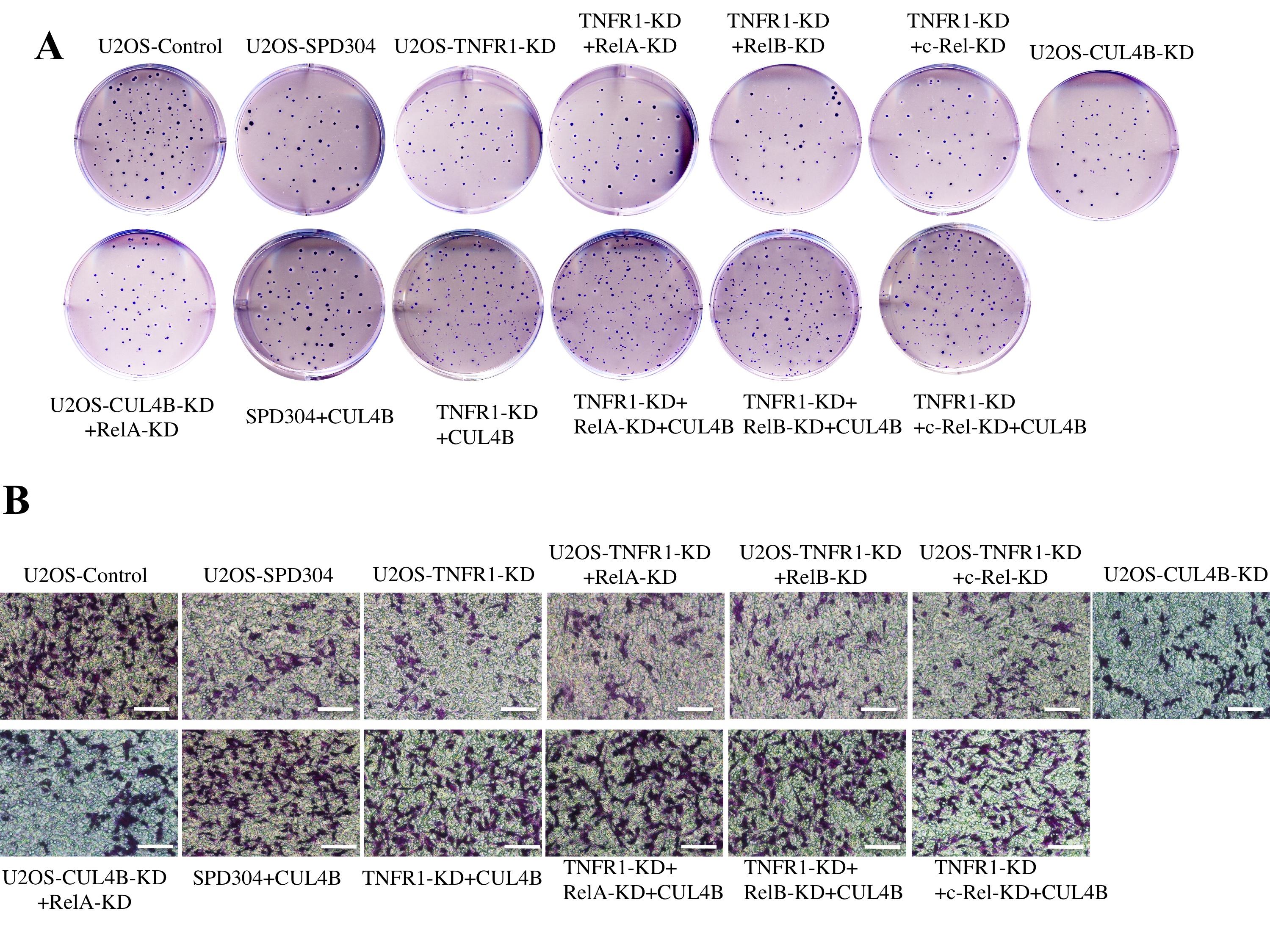


**Supplementary Figure 9. Disruption of TNF-α/NF-κB axis decreases colony formation rates and cell invasion.**

1. Disruption of the TNF-α/NF-κB axis inhibited osteosarcoma cell colony formation rates. U2OS cells transfected with Con-shRNA (Control); U2OS cells treated with SPD304; U2OS cells knocked down for TNFR1 (TNFR1-KD), CUL4B (CUL4B-KD), both TNFR1 and RelA (TNFR1-KD+RelA-KD), TNFR1 and RelB (TNFR1-KD+RelB-KD), TNFR1 and c-RelA (TNFR1-KD+c-RelA-KD), or RelA and CUL4B (RelA-KD+CUL4B-KD); and the above cells overexpressing *CUL4B*, including SPD304+CUL4B, TNFR1-KD+CUL4B, TNFR1-KD+Rel-A+CUL4B, TNFR1-KD+Rel-B+CUL4B, and TNFR1-KD+c-Rel+CUL4B were seeded onto 12-well plates and cultured with 0.1 ml of fresh medium containing 0.5% FBS for two weeks before pictures were taken. **(B)** Disruption of the TNF-α/NF-κB axis decreased cell invasiveness. Cells used in (A) were subjected to the Boyden chamber assays, and the invasive cells were photographed.
2.
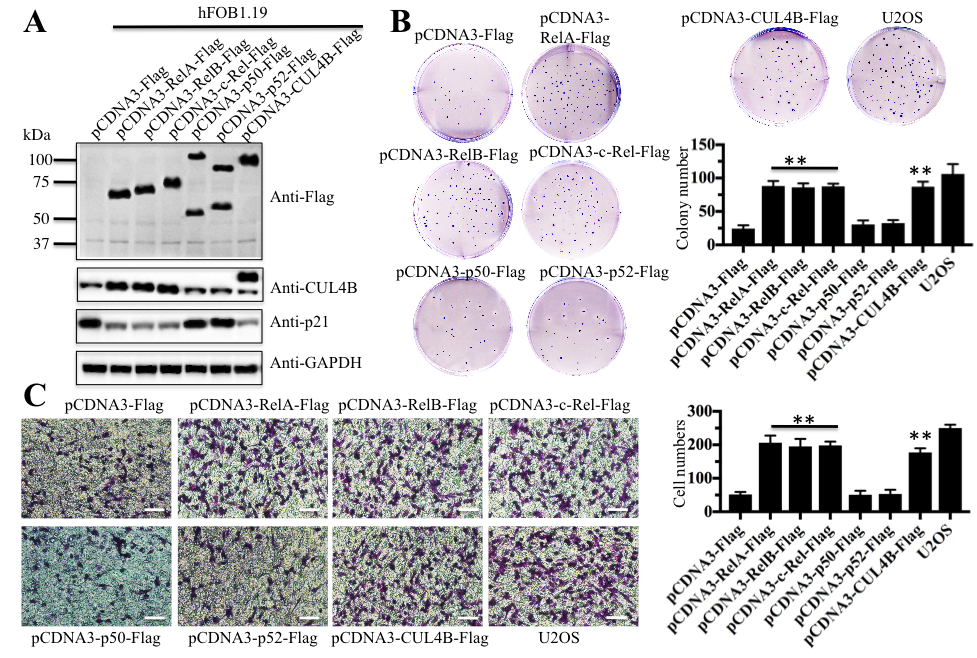
**Supplementary Figure 10. Overexpression of *RelA*, *RelB*, *c-Rel*, or *CUL4B* in hFOB1.19 cells results in effects similar to those in U2OS cells.**

**(A)** Overexpression of *RelA*, *RelB*, *c-Rel*, or *CUL4B* in hFOB1.19 cells decreased p21 protein levels. hFOB1.19 cells were transfected with pCDNA3-Flag, pCDNA3-RelA-Flag, pCDNA3-RelB-Flag, pCDNA3-c-Rel-Flag, pCDNA3-p50-Flag, pCDNA3-p52-Flag, or pCDNA3-CUL4B-Flag plasmids. The resulting cells were subjected to Western blots to examine Flag-tagged proteins and p21. GAPDH was used as a loading control. **(B)** Overexpression of *RelA*, *RelB*, *c-Rel*, and *CUL4B* in hFOB1.19 cells enhanced colony formation ability. Cells used in (A) were seeded onto 12-well plates, and the cells were cultured with 0.1 ml of fresh medium containing 0.5% FBS for two weeks before pictures were taken. **(C)** Overexpression of *RelA*, *RelB*, *c-Rel*, and *CUL4B* in hFOB1.19 cells increased cell invasion. Cells used in (A) were subjected to the Boyden chamber assays, and the invasive cells were photographed.


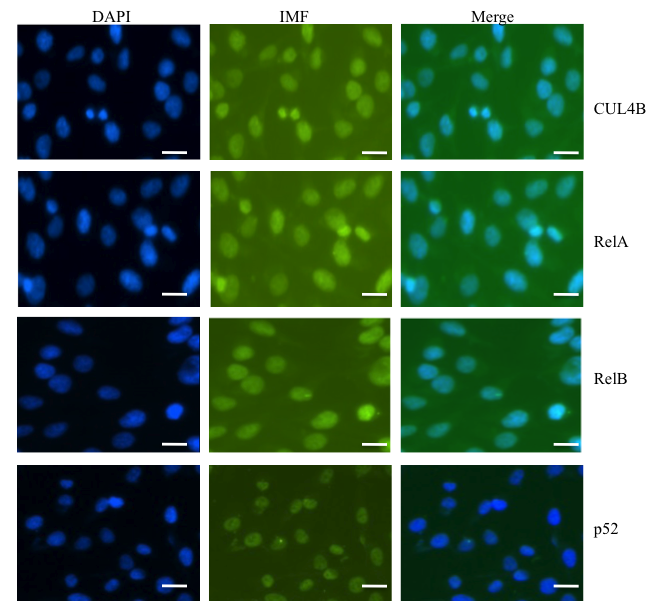


**Supplementary Figure 11. NF-κB subunits and CUL4B were localized in the nucleus in a melanoma cell line.**

The human melanoma cell line A375 was used for immunofluorescence (IMF) staining with anti-CUL4B, anti-RelA, anti-RelB, and anti-p52 antibodies. The nuclei are counterstained with DAPI (blue, left panel). The localization and levels of CUL4B, RelA, RelB, and p52 protein are shown as green (middle panel), and the merged images are in the right panel. Bars = 25 µm.


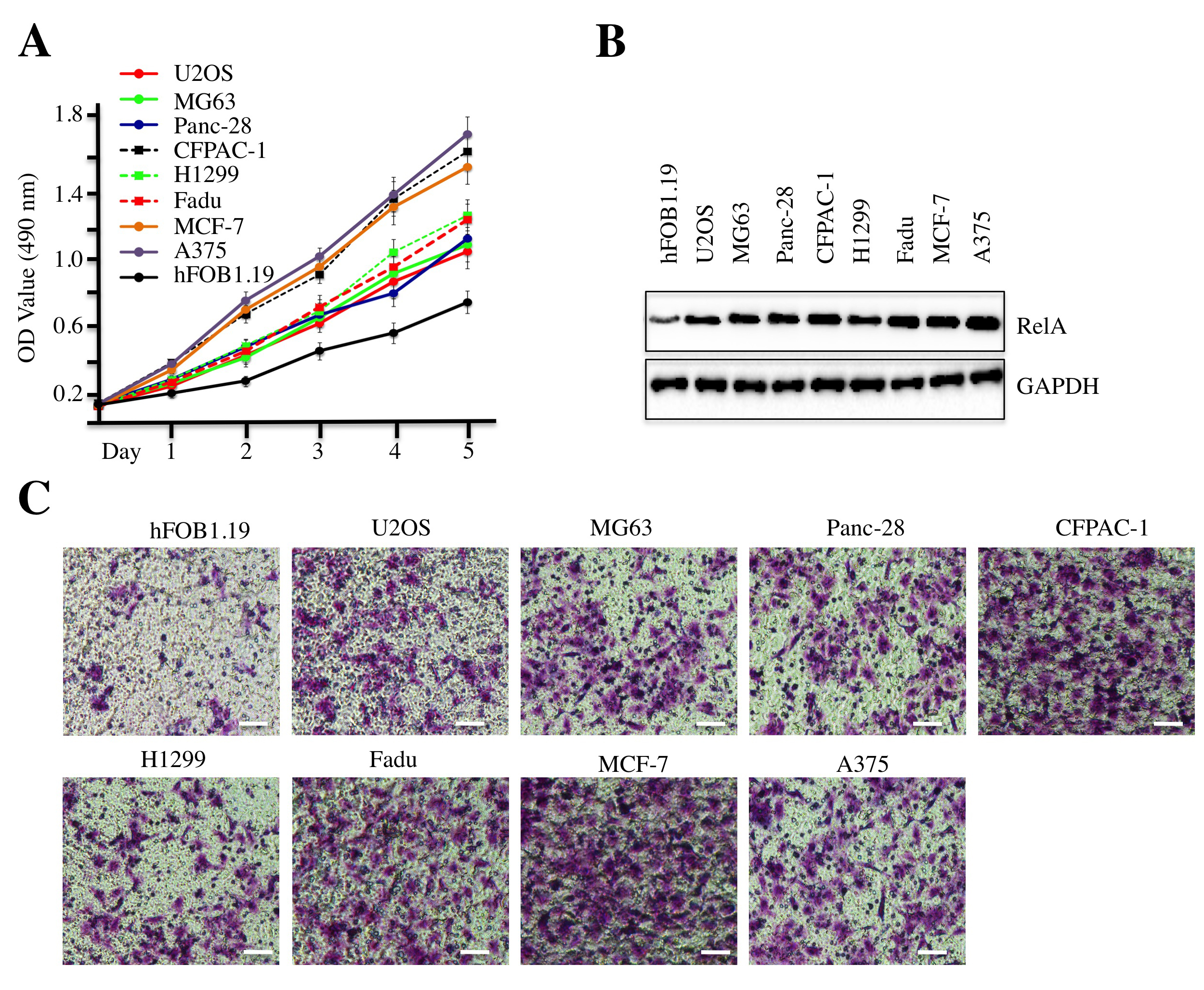


**Supplementary Figure 12. Cell growth and invasion in different cell types.**

The hFOB1.19, U2OS, MG63, two pancreatic adenocarcinoma cell lines including Panc-28 and CFPAC-1, H1299 (a lung cancer cell line), MCF-7 (a breast cancer cell line), Fadu (a carcinoma cell line) and A375 cells were subjected into cell proliferation assay **(A)**. RelA protein levels were also determined **(B)**. **(C)** Cells used in (A) were subjected to the Boyden chamber assays, and the invasive cells were photographed.


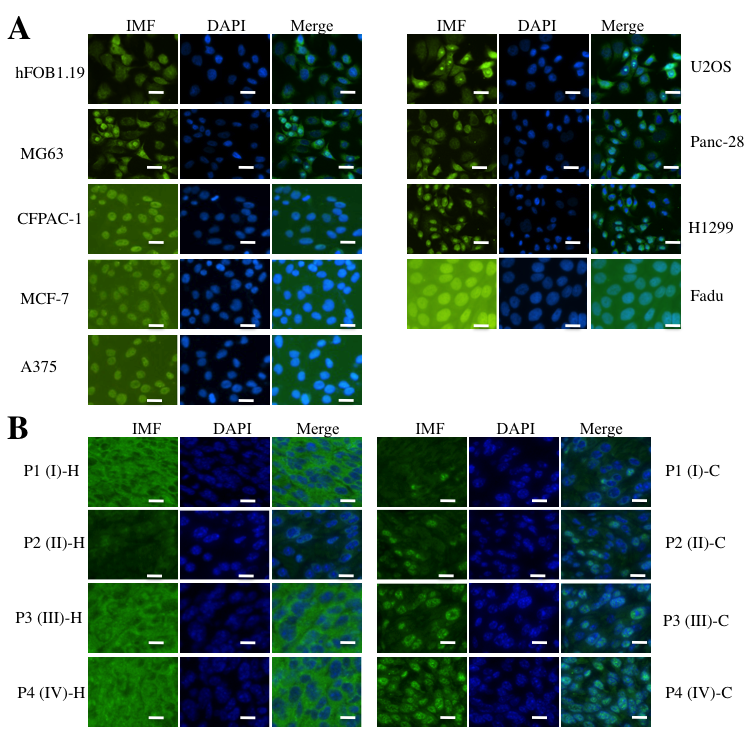


**Supplementary Figure 13. Different cancer cell lines exhibited different nuclear levels of RelA.**

**(A)** The hFOB1.19, U2OS, MG63, Panc-28, CFPAC-1, H1299, MCF-7, Fadu and A375 cells were subjected into IMF staining with anti-RelA antibody. **(B)** The non-cancerous and cancerous samples from 4 different patients who underwent different MSTS stages (from I to IV), were subjected to IMF staining with anti-RelA antibody. The localization and levels of RelA protein are shown as green (left panel). The nuclei are counterstained with DAPI (blue, middle panel). The merged images are in the right panel. Bars = 25 µm.

**Supplementary Table-1. siRNA and shRNA information**

| Gene | Category | Company | Catalog Number |
| --- | --- | --- | --- |
| RelA | siRNA | Sigma | NM_021975 |
| RelA | shRNA | Sigma | TRCN0000353629 |
| RelA | shRNA | Sigma | TRCN0000329802 |
| RelB | siRNA | Sigma | NM_006509 |
| RelB | shRNA | Sigma | TRCN0000014717 |
| RelB | shRNA | Sigma | TRCN0000014713 |
| c-Rel | siRNA | Sigma | NM_002908 |
| c-Rel | shRNA | Sigma | TRCN0000039984 |
| c-Rel | shRNA | Sigma | TRCN0000039986 |
| p50 | shRNA | Sigma | TRCN0000006521 |
| p52 | shRNA | Sigma | TRCN0000356047 |
| CUL4B | siRNA | Sigma | NM_001079872 |
| CUL4B | shRNA | Sigma | TRCN0000006532 |
| CUL4B | shRNA | Sigma | TRCN0000006536 |
| TNFR1 | shRNA | Sigma | TRCN0000378351 |
| TNFR1 | shRNA | Sigma | TRCN0000359597 |

**Supplementary Table-2. The clinicopathological futures of 54 osteosarcoma patients and miR-300 expression**

|  |  | **Relative expression of *CUL4B*** | |  |
| --- | --- | --- | --- | --- |
| **Characteristics** | **Number of cases** | **Low (log2<0)** | **High (log2**≥**0)** | ***P-*value** |
| **Gender** |  |  |  |  |
| Male  Female | 27  27 | 5  7 | 22  20 | 0.7782 |
| **Age**  ≥20  <20  **Tumor size**  ≥12 cm  <12  **MSTS stages**  I  II/III/IV | 12  42  15  39  16  38 | 3  9  0  12  16  0 | 9  33  15  27  0  38 | 0.7548  0.0026*  <0.0001** |

*P < 0.05, **P < 0.001
